# ANKRD55 is a key regulator of T cell inflammation in multiple sclerosis

**DOI:** 10.1172/JCI195214

**Published:** 2025-10-15

**Authors:** Chuyu Wu, Meiling Jiang, Xue Yang, Yixuan Liu, Bin Huang, Yi Guo, Runjing Cao, Zhihui Cui, Guozhen Deng, Weiyan Wang, Mengdi Guo, Zhiyong Lin, Jiahui Fan, Lin-ming Zhang, Lorenzo Di Cesare Mannelli, Tao Pang, Chenhui Wang, Cun-Jin Zhang

**Affiliations:** 1 Department of Neurology, Sichuan Provincial People’s Hospital, School of Medicine, University of Electronic Science and Technology of China, Chengdu, China.; 2 Department of Science and Technology, Sichuan Provincial People’s Hospital, University of Electronic Science and Technology of China, Chengdu, China.; 3 Center for Rehabilitation Medicine, Department of Neurology, Zhejiang Provincial People’s Hospital, Affiliated People’s Hospital, Hangzhou Medical College, Hangzhou, China.; 4 The Key Laboratory for Human Disease Gene Study of Sichuan Province and the Department of Laboratory Medicine, Sichuan Provincial People’s Hospital, University of Electronic Science and Technology of China, Chengdu, China.; 5 Research Unit for Blindness Prevention of the Chinese Academy of Medical Sciences, Sichuan Academy of Medical Sciences and Sichuan Provincial People’s Hospital, Chengdu, China.; 6 Sichuan Medical Laboratory Clinical Medical Research Center, Sichuan Provincial People’s Hospital, Chengdu, China.; 7 Department of Neurology, The First Affiliated Hospital of Kunming Medical University, Kunming, China.; 8 Department of Neurosciences, Psychology, Drug Research and Child Health (NEUROFARBA), Section of Pharmacology and Toxicology, University of Florence, Florence, Italy.; 9 State Key Laboratory of Natural Medicines, New Drug Screening and Pharmacodynamics Evaluation Center, Key Laboratory of Drug Quality Control and Pharmacovigilance (Ministry of Education), China Pharmaceutical University, Nanjing, China.

**Keywords:** Autoimmunity, Immunology, Autoimmune diseases, Multiple sclerosis, T cell receptor

## Abstract

Multiple sclerosis (MS) is a progressive, chronic, and highly disabling neuroinflammatory disorder characterized by demyelination and T cell–driven inflammation. Pathogenic T cells play a central role in MS, but effective therapeutic targeting remains challenging. Here, we identified ankyrin repeat domain–containing protein 55 (ANKRD55) as a key regulator of T cell function by single-cell transcriptomic analysis of cerebrospinal fluid and blood from MS patients. *ANKRD55* was predominantly expressed in CD4^+^ T cells in both compartments. Genetic ablation of *Ankrd55* led to a robustly reduced disease severity and neuroinflammation in experimental autoimmune encephalomyelitis (EAE), a widely used animal model for MS. Furthermore, T cell–specific deficiency of *Ankrd55* significantly impaired Th1 polarization and Th17 differentiation, reducing EAE pathogenicity. Mechanistically, we found that *Ankrd55* deficiency disrupted T cell receptor (TCR) signaling integrity. We demonstrated that ANKRD55 regulates the formation of the immune synapse, an essential prerequisite for TCR activation, by interacting with subunits of the chaperonin-containing TCP1 (CCT) complex and modulating its activity, enhancing its assembly by competing with CCT5 for binding to TCP1, CCT3, and CCT6. This facilitates proper microtubule organization and TCR activation. These findings establish ANKRD55 as a critical regulator of TCR signaling and highlight its therapeutic potential in pathogenic T cell–driven autoimmune diseases.

## Abstract 2

<a>ANKRD55 promotes CD4+ T cell activation in Multiple Sclerosis by facilitating the assembly of CCT complexes at the immune synapse</a>

## Graphical abstract

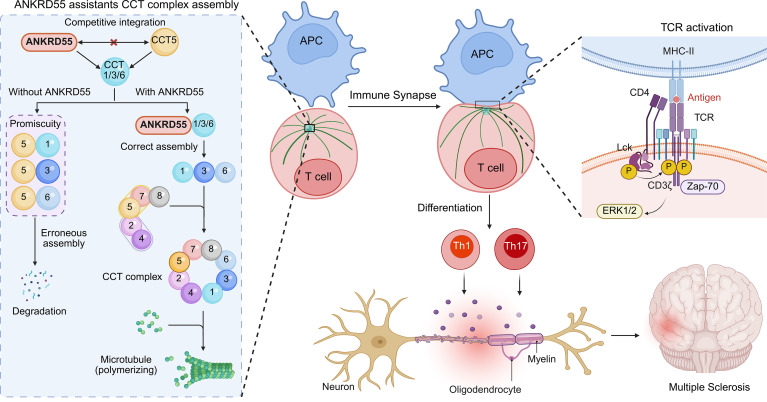

## Introduction

Multiple sclerosis (MS), is a severe autoimmune disorder, mainly affects the CNS and is characterized by inflammatory infiltration, demyelination, and axonal damage in the brain and the spinal cord (1, 2). Experimental autoimmune encephalomyelitis (EAE), a well-established experimental model for MS, has been extensively studied to elucidate the underlying mechanisms of MS (3). MS is a highly debilitating and fatal disease that predominantly affects young and middle-aged populations, with a particularly high prevalence in individuals aged 20–40. The exact cause of MS is not yet known, but it is believed to be a genetically complex disease that results from a combination of environmental factors, such as low levels of vitamin D (4), infection with EBV (5–7), smoking (8), premature obesity (9), and genetic predispositions (10, 11).

T cells are widely recognized as central mediators of MS pathogenesis and key regulators of CNS-directed autoimmunity (12). Once activated, autoreactive T cells initiate a cascade of immune events characteristic of MS. Activated T cells can traverse the blood-brain barrier and enter the CNS, where they further activate resident immune cells within the CNS (13). The migration of peripheral immune cells into the CNS is driven by a coordinated process involving T cell activation in peripheral lymphoid tissues and upregulation of chemokine receptors and adhesion molecules (14). Upon entering the CNS, autoreactive T cells, along with other infiltrating immune cell types (including B cells and macrophages), target myelin, leading to inflammation and demyelination of axons. Activated T cells facilitate the release of proinflammatory cytokines, such as IFN-γ and TNF-α, which exacerbate inflammation within the CNS. These cytokines recruit additional immune cells, amplify the immune response, and contribute to the damage of the myelin sheath and the axonal injury. Although CD4^+^ T cells, CD8^+^ T cells, and γδ T cells all play pivotal roles in the pathogenesis of MS (13, 15), CD4^+^ T cells exhibit the most pronounced effect (16, 17). However, it remains challenging to target CD4^+^ T cells therapeutically to suppress the autoimmune inflammation.

Extensive research has revealed that the pathogenesis of MS may involve T cells misidentifying myelin as an antigen (18, 19). The T cell receptor (TCR) and the immune synapse are central to the immunopathogenesis of MS, playing pivotal roles in the activation and autoreactive behavior of T cells that drive the autoimmune response. The TCR is a cell surface receptor expressed on T cells that specifically recognizes processed antigenic peptides presented on MHC molecules by antigen-presenting cells (APCs) (20). The interaction between the TCR and the MHC-peptide complex is the key signal for T cell activation. When the TCR binds to its specific antigen-MHC complex, it initiates intracellular signaling events that drive T cell activation. These signaling pathways enable T cell migration toward APCs to establish a stable contact interface, known as the immune synapse (21–23). As an essential prerequisite for TCR activation, the immune synapse represents a highly organized structure that forms at the interface between a T cell and an APC. Following initial contact with the APC, T cells rapidly expand and stabilize the interaction through cytoskeletal remodeling–mediated deformation, ultimately forming the immune synapse. The T cell–APC interaction facilitates reorganization of the cytoskeleton in the T cell (24, 25) and cytokine release to propagate the immune response.

MS is closely associated with dysregulated T cell responses (15). Specifically, Th1 and Th17 subsets of CD4^+^ T cells are key drivers of inflammation and demyelination (26, 27). The immune synapse is critical in the differentiation of T cells into these subsets (28), which produce cytokines that initiate and sustain the inflammatory cascade in MS. T cell activation through the immune synapse leads to a series of downstream effects, including T cell proliferation, cytokine secretion and effector function. Beyond activation, the immune synapse plays a pivotal role in regulating the immune response by ensuring precise spatial organization of molecules within the synapse, thereby enabling the T cell to receive accurate signaling cues (28). Disruption of immune synapse formation or its associated signaling processes can impair the immune response, resulting in conditions such as autoimmunity, immunodeficiency, or inadequate immune responses.

Although the central role of inflammatory T cells in the pathogenesis of MS is widely recognized, how T cell inflammation could be targeted therapeutically remains unclear. Herein, we identified ankyrin repeat domain–containing protein 55 (ANKRD55) as a key regulator of T cell functions using single-cell transcriptomic analysis of the cerebrospinal fluid (CSF) and blood from MS patients. Genetic or T cell–specific ablation of *Ankrd55* led to a robustly reduced disease severity and neuroinflammation in EAE. In addition, we found that ANKRD55 is predominantly expressed in CD4^+^ T cells in both MS patients and EAE mice. Deficiency of *Ankrd55* significantly impaired Th1 polarization and Th17 differentiation. Mechanistically, we demonstrated that ANKRD55 regulates the formation of the immune synapse, an essential prerequisite for TCR activation. Specifically, ANKRD55 interacts with subunits of the chaperonin-containing TCP1 (CCT) complex and modulates its activity, enhancing the assembly of the CCT complex, which is necessary for tubulin polymerization and microtubule dynamics. These findings establish ANKRD55 as a critical regulator of TCR signaling and highlight its potential as a promising therapeutic target for pathogenic T cell–driven autoimmune diseases.

## Results

### ANKRD55 promotes disease progression in MOG_35–55_-induced EAE.

**Figure 1:**
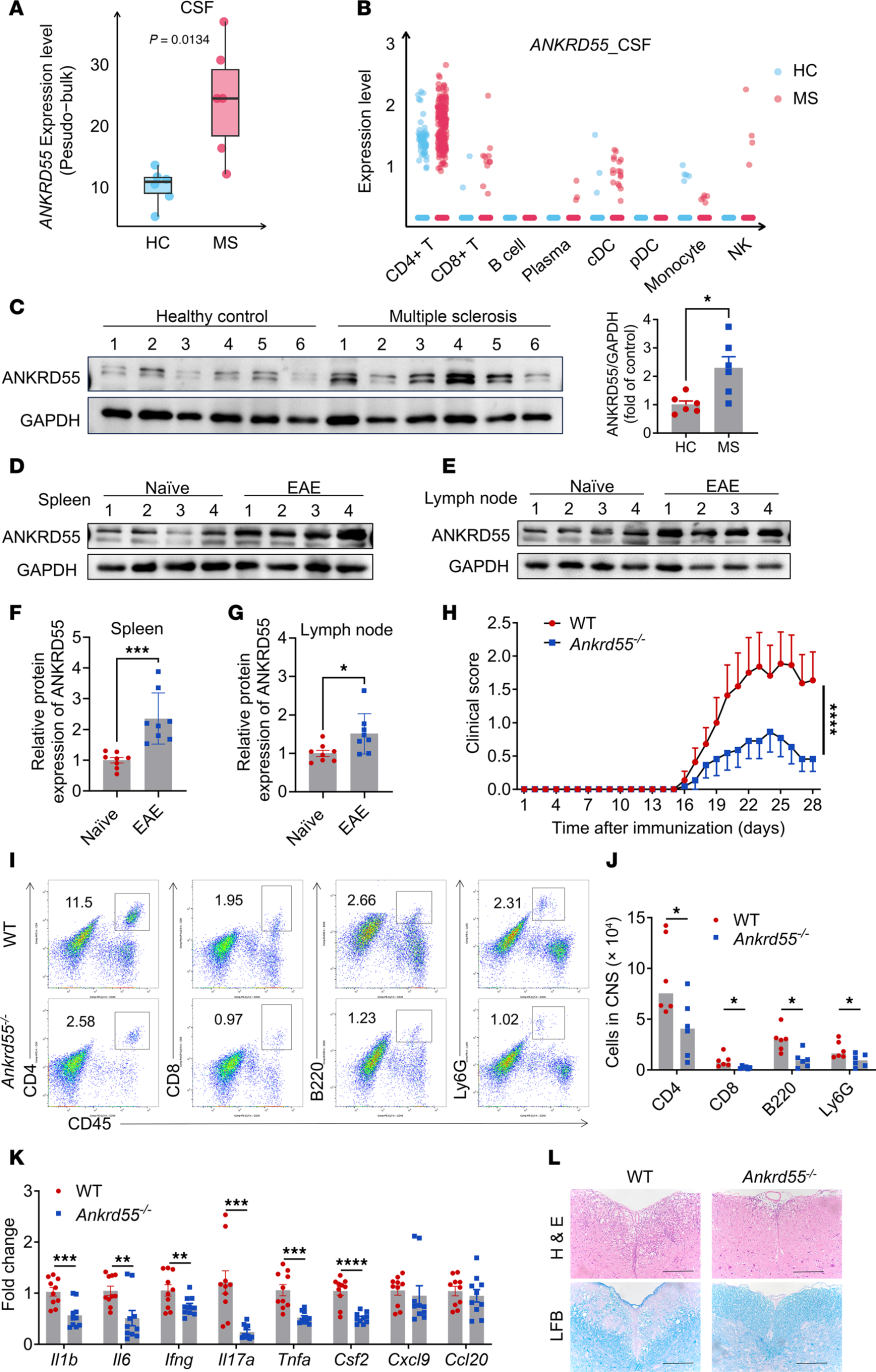
ANKRD55 promotes disease progression of MOG_35–55_-induced EAE. (**A**) Pseudo-bulk analysis of *ANKRD55* expression in CSF cells from healthy controls (HC) and MS patients; each dot represents an individual sample. (**B**) The expression level of *ANKRD55* was measured at single-cell resolution in diverse cells within CSF samples; each dot represents one cell. (**C**) Immunoblot analysis was performed to compare ANKRD55 expression between PBMCs from healthy human control subjects and MS patients. (**D**–**G**) Immunoblot analysis of ANKRD55 in spleen (**D**) and lymph node (**E**) of naive and EAE mice. Densitometric quantification of band intensities was performed using ImageJ, and protein expression levels were normalized to GAPDH (**F** and **G**). (**H**) The mean clinical score of EAE was assessed in WT and *Ankrd55*^–/–^ mice (*n* = 11 mice per group) following active immunization with MOG_35–55_ peptide. (**I** and **J**) Infiltrated immune cells in the brain were analyzed by flow cytometry in WT and *Ankrd55*^–/–^ mice at the peak of EAE disease. Data are presented as representative plots (**I**) and quantification of cells in the CNS (**J**). (**K**) Quantitative analysis of inflammatory cytokine and chemokine expression in the spinal cords of WT and *Ankrd55*^–/–^ mice at the peak of EAE. (**L**) Representative histological images of spinal cord sections from WT and *Ankrd55*^–/–^ mice at peak disease. LFB, Luxol fast blue. Scale bars: 200 μm. *P < 0.05, **P < 0.01, ***P < 0.001, ****P < 0.0001, based on unpaired, 2-tailed *t* test (**A**, **C**, **F**, **G**, **J**, and **K**) or 2-way ANOVA with Tukey’s multiple-comparison test (**H**). Data are shown as mean ± SEM.

Although the central role of pathogenic T cells in MS is well established, precise modulation of these T cells remains a significant therapeutic challenge. To identify key regulators controlling T cell inflammation in MS pathogenesis, we analyzed single-cell transcriptomic data (GSE138266; Gene Expression Omnibus [GEO] database) from CSF of MS patients, as published and shared by Schafflick et al. (29). Our findings revealed that *ANKRD55* levels were elevated in MS patients compared with healthy controls (Figure 1, A and B).

We further investigated ANKRD55 expression in PBMCs of MS patients using immunoblotting analysis, which demonstrated significantly increased ANKRD55 levels in PBMCs from MS patients compared with healthy controls (Figure 1C). Additionally, we examined ANKRD55 expression in the spleen and lymph nodes of mice with MOG_35–55_-induced EAE. Our results showed that ANKRD55 expression was notably higher in these tissues of EAE model mice compared with naive mice (Figure 1, D–G). These findings suggest that ANKRD55 may play a critical role in the pathogenesis of both MS and EAE.

To directly evaluate the necessity of ANKRD55 for EAE progression, we generated *Ankrd55*-KO mice (Supplemental Figure 1A; supplemental material available online with this article; https://doi.org/10.1172/JCI195214DS1). Using flow cytometry, we analyzed cell distribution in the spleen and lymph nodes of both naive WT and *Ankrd55*-KO mice and observed no significant differences in cell types (Supplemental Figure 1, C–J). Furthermore, we compared disease severity between WT and *Ankrd55*-deficient (*Ankrd55*^–/–^) mice. Our results indicated that *Ankrd55*^–/–^ mice exhibited significantly reduced disease severity compared with WT mice (Figure 1H). Inflammatory cell infiltration, including CD4^+^ T cells, B cells, and neutrophils, was also significantly reduced in *Ankrd55*^–/–^ mice (Figure 1, I and J). Additionally, expression of inflammatory cytokines and chemokines in the spinal cord of *Ankrd55*^–/–^ mice was notably decreased (Figure 1K). Consistently, histological analysis of spinal cords at the peak of disease revealed that *Ankrd55*^–/–^ mice exhibited fewer infiltrating inflammatory cells and attenuated demyelination compared with WT mice (Figure 1L). These findings collectively demonstrate that deletion of ANKRD55 substantially protects mice from EAE pathogenesis, with a notable reduction in both disease severity and associated histopathological features.

### ANKRD55 is predominantly expressed in CD4^+^ T cells and is associated with T cell development.

**Figure 2:**
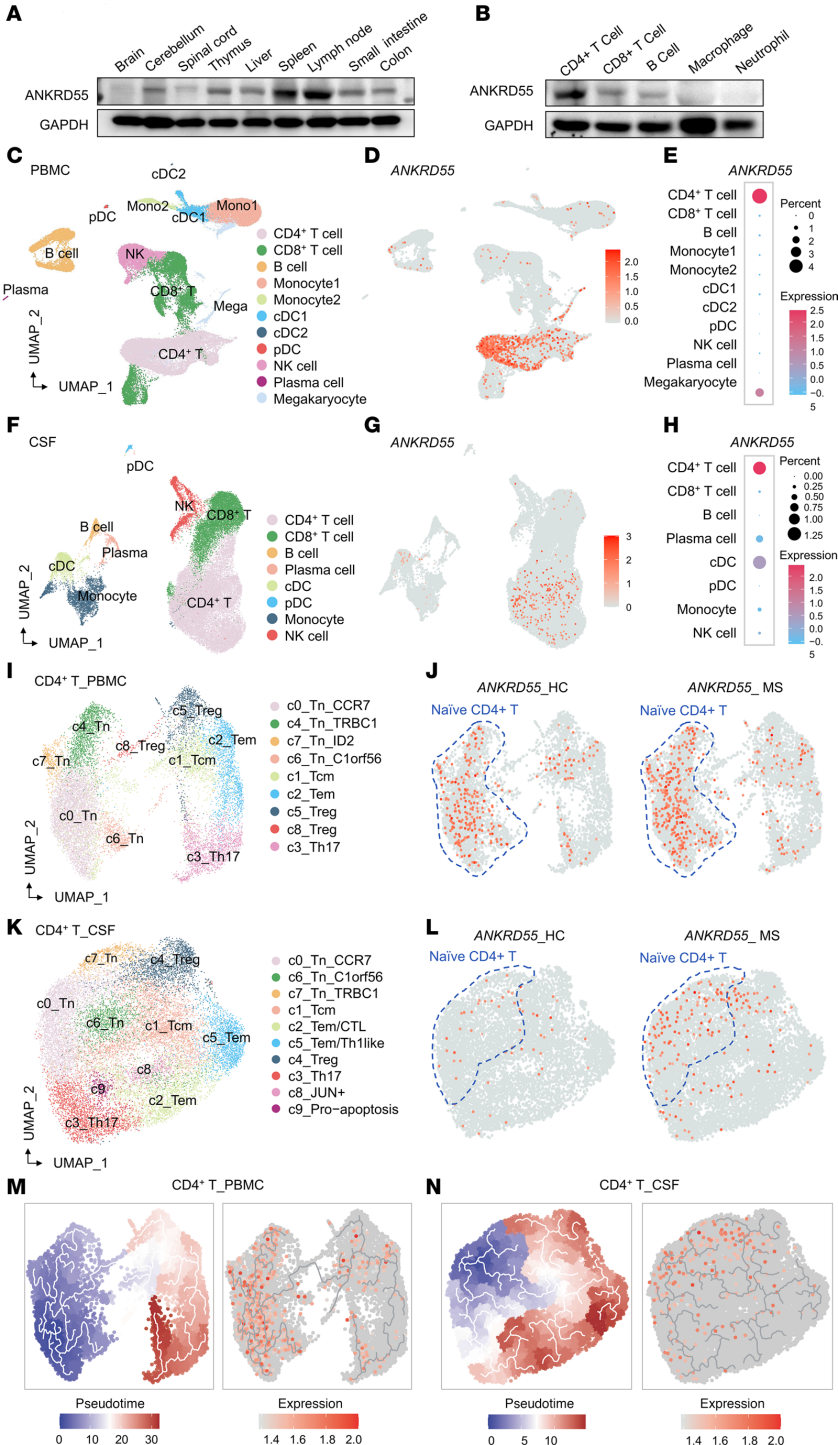
ANKRD55 is mainly expressed in CD4^+^ T cells and is engaged in T cell development. (**A**) Immunoblot analysis of ANKRD55 expression was performed in various tissues (brain, cerebellum, spinal cord, thymus, liver, spleen, lymph node, and gastrointestinal tract) from WT mice. (**B**) Immunoblot analysis of ANKRD55 expression was conducted in different immune cell populations (CD4^+^ T cells, CD8^+^ T cells, B cells, macrophages, and neutrophils) within WT mouse PBMCs. (**C**) Uniform Manifold Approximation and Projection (UMAP) visualization of 11 distinct subclusters of PBMCs. (**D**) UMAP visualization showing the distribution of *ANKRD55*-expressing cells (red dots) in PBMCs. (**E**) A bubble plot was generated to depict the expression level of *ANKRD55* in individual cells within PBMC populations. (**F**) UMAP visualization of 8 distinct subclusters of CSF cells. (**G**) UMAP visualization showing the distribution of *ANKRD55*-expressing cells (red dots) in CSF cells. (**H**) A bubble plot was generated to show the expression levels of *ANKRD55* in different CSF cell populations. (**I**) UMAP plot showing subclustering of CD4^+^ T cells from PBMCs. Cluster names were manually assigned. (**J**) UMAP visualization showing the distribution of *ANKRD55*-expressing cells (red dots) in CD4^+^ T cells from PBMCs. Naive CD4^+^ T cell clusters are outlined with blue dashed lines to highlight *ANKRD55* enrichment in these regions. (**L**) UMAP visualization showing the distribution of *ANKRD55*-expressing cells (red dots) in CD4^+^ T cells from PBMCs. Naive CD4^+^ T cell clusters are outlined with blue dashed lines to highlight *ANKRD55* enrichment in these regions. (**M** and **N**) Pseudotime trajectory analysis of CD4^+^ T cells inferred using Monocle 2 from PBMCs (**M**) and CSF (**N**), with cells colored by pseudotime values (blue to red), reflecting progression from naive to more differentiated states.

Having identified that ANKRD55 promotes EAE progression, we next focused on ANKRD55 expression and cellular functions. We first investigated ANKRD55 expression across various tissues implicated in EAE progression, focusing on both peripheral immune organs (such as the spleen and lymph nodes) and the CNS. Our findings revealed that ANKRD55 is primarily expressed in the spleen and lymph nodes (Figure 2A and Supplemental Figure 8A).

We further examined ANKRD55 expression in specific peripheral immune cell populations. We sorted different immune cells from mouse spleen and analyzed ANKRD55 protein levels by Western blotting. Our results revealed that ANKRD55 protein levels were significantly higher in CD4^+^ T cells compared with other immune cell types in mice (Figure 2B and Supplemental Figure 8B). This observation aligns with our analysis of single-cell RNA-Seq data from PBMCs and CSF of MS patients, which also indicates elevated *ANKRD55* expression levels in CD4^+^ T cells (Figure 1B, Figure 2, C–H, and Supplemental Figure 2, A and B). By stratifying CD4^+^ T cells into distinct subpopulations, we found that *ANKRD55* is predominantly expressed in naive CD4^+^ T cells (Figure 2, I–L, and Supplemental Figure 2, C and D). Pseudotime analysis revealed that *ANKRD55* expression occurs early during CD4^+^ T cell differentiation (Figure 2, M and N). These results suggest that *ANKRD55* is predominantly expressed in CD4^+^ T cells and may play a critical role in the early stages of CD4^+^ T cell differentiation.

### ANKRD55 is essential for promoting Th1 and Th17 differentiation.

**Figure 3:**
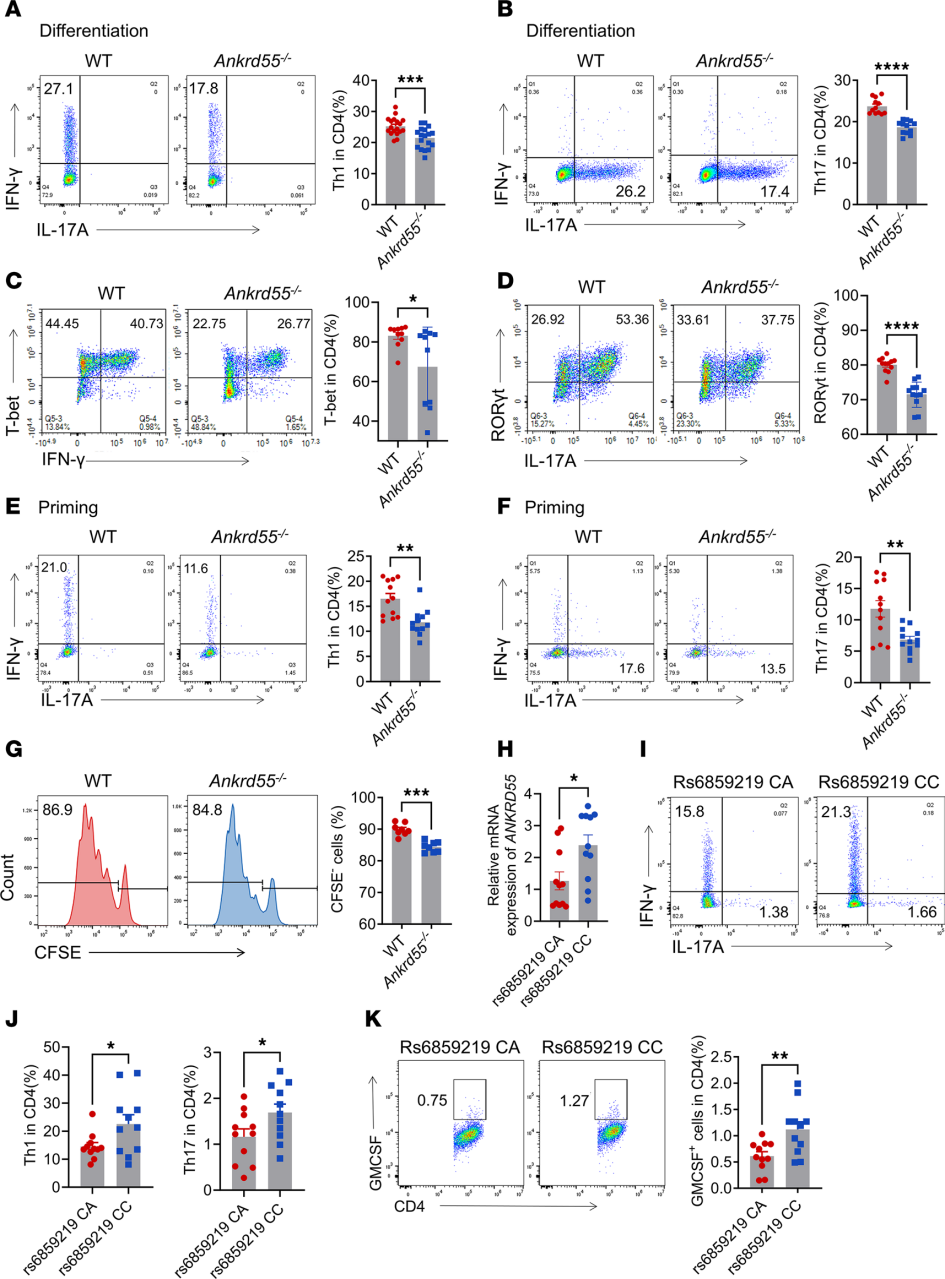
ANKRD55 critically promotes Th1 and Th17 differentiation. (**A** and **B**) Flow cytometry analysis of naive CD4^+^ T cells sorted by magnetic beads was performed. The cells were cultured on anti-CD3/CD28–coated plates under Th1 (*n* = 18) or Th17 (*n* = 12) polarization conditions. (**C** and **D**) Naive CD4^+^ T cells were isolated from WT and *Ankrd55*^–/–^ mice and cultured under Th1- or Th17-polarizing conditions for 4 days. Expression of T-bet (**C**) (under Th1 conditions) and RORγt (**D**) (under Th17 conditions) was evaluated by intracellular staining followed by flow cytometry. Representative flow cytometry plots (left) and quantification of T-bet^+^ and RORγt^+^ cells (right) are shown (*n* = 10 or 12). (**E** and **F**) Cells isolated from the spleens of mice on day 10 after immunization were restimulated with MOG_35–55_ and induced to polarize toward Th1 or Th17, followed by intracellular staining for IL-17A and IFN-γ and analysis by flow cytometry (*n* = 12). (**G**) CD4^+^ T cells were sorted from mouse spleens using magnetic beads. The cells were labeled with CFSE (0.5 μM at 37°C for 5 minutes), and the proportion of proliferating cells was analyzed by flow cytometry after culturing for 48 hours on plates coated with anti-CD3 and anti-CD28 (*n* = 8). (**H**) Human PBMCs were divided into different groups based on single nucleotide polymorphism genotype of *ANKRD55*. Expression of *ANKRD55* mRNA was detected by RT-qPCR (*n* = 11). (**I**–**K**) The proportion of CD4^+^ T cells secreting IFN-γ, IL-17A, and GM-CSF in PBMCs was analyzed by flow cytometry (*n* = 11). (**I** and **J**) IFN-γ and IL-17A; (**K**) GM-CSF. *P < 0.05, **P < 0.01, ***P < 0.001, ****P < 0.0001, based on unpaired, 2-tailed *t* test. Data are shown as mean ± SEM.

Autoimmune conditions, particularly MS, are closely associated with the differentiation of Th1 and Th17 cells. Therefore, we investigated the impact of ANKRD55 on the differentiation of Th1 and Th17 cells. Our data showed no significant alterations in the ratio of Th1 to Th17 cells in the spleen and lymph node of untreated *Ankrd55*^–/–^ mice in vivo (Supplemental Figure 3, A–D). However, naive CD4^+^ T cells from *Ankrd55*^–/–^ mice exhibited impaired Th1 and Th17 cell polarization compared with WT mice (Figure 3, A and B). *Ankrd55* KO also resulted in a significant reduction in the expression of both T-bet and RORγt (Figure 3, C and D), suggesting that ANKRD55 positively regulates the transcriptional programs associated with Th1 and Th17 differentiation. Additionally, we investigated whether ANKRD55 contributes to the priming of MOG-reactive T cell populations. Splenocytes isolated from EAE mice (day 10) were stimulated with MOG in exposure to IL-12 (Th1 polarization) or IL-23 (Th17 polarization), and we found that splenocyte differentiation into Th1 and Th17 cells was significantly inhibited in *Ankrd55*^–/–^ mice in vitro (Figure 3, E and F).

Additionally, we assessed the proliferative capacity of CD4^+^ T cells using CFSE dye following stimulation with CD3 and CD28 antibodies. Our results indicated that CD4^+^ T cells from *Ankrd55*^–/–^ mice exhibited reduced proliferative ability compared with WT mice (Figure 3G). Furthermore, we evaluated the impact of *Ankrd55* deletion on T cell activation by stimulating purified CD4^+^ and CD8^+^ T cells with anti-CD3/CD28 antibodies and assessing surface expression of CD69 (Supplemental Figure 3, G and H), a marker for early T cell activation, by flow cytometry. We observed that *Ankrd55* deficiency impaired activation of both CD4^+^ and CD8^+^ T cells, with a more pronounced effect on CD4^+^ T cells. Genome-wide association studies have linked the C allele of rs6859219 in *ANKRD55* to an increased risk of developing MS (30–32). We collected PBMCs from healthy volunteers and identified individuals carrying the rs6859219 mutation. Reverse transcription–quantitative PCR (RT-qPCR) analysis revealed that PBMCs from volunteers harboring more C alleles exhibited elevated *ANKRD55* expression levels (Figure 3H). We further examined the proportions of Th1 and Th17 cells in PBMCs and observed a notably higher proportion of them in individuals carrying the C allele (Figure 3, I and J). Additionally, we found that CD4^+^ T cells from volunteers with the rs6859219 (C/C) genotype exhibited increased levels of GM-CSF, an inflammatory Th17 signature cytokine, compared with those with the C/A genotype (Figure 3K). These results further suggest that ANKRD55 is involved in the differentiation of Th1 and Th17 cells in both humans and the EAE mouse model.

### Genetic deletion of ANKRD55 in T cells protects from both active and Th1/Th17-mediated EAE.

**Figure 4:**
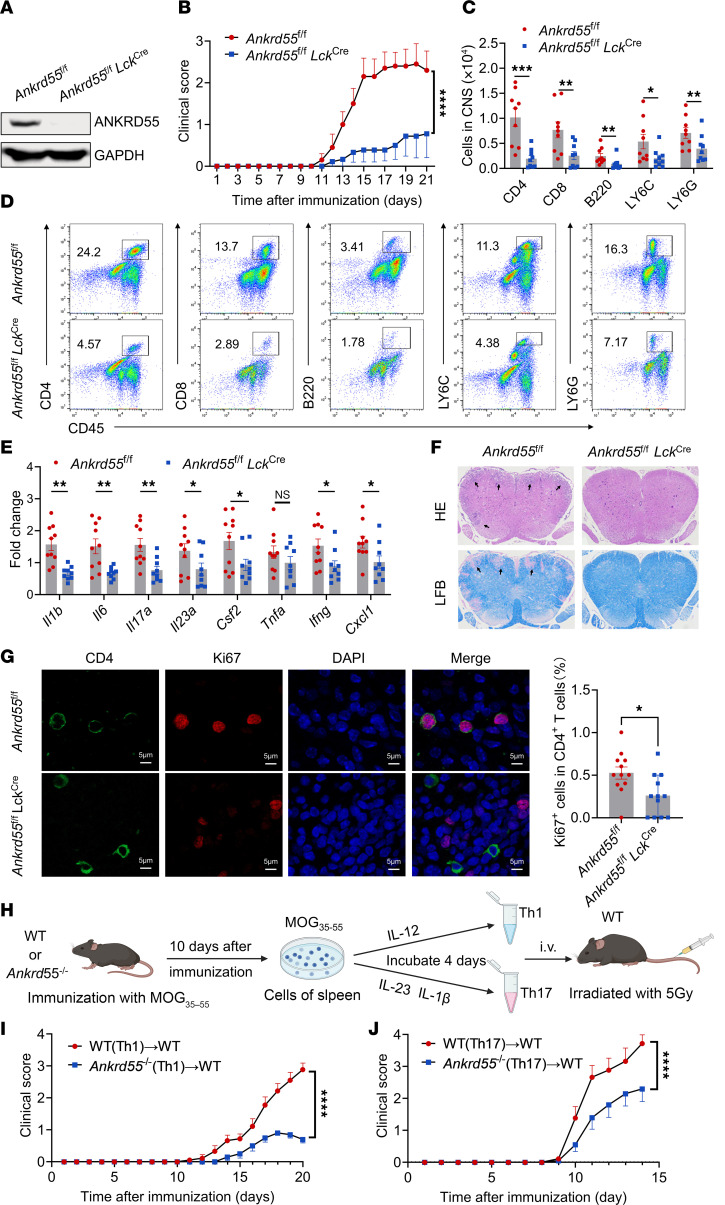
Genetic deletion of *Ankrd55* in T cells protects from both active and Th1/Th17-mediated EAE. (**A**) Immunoblot analysis of ANKRD55 protein expression in CD4^+^ T cells from *Ankrd55^fl/fl^* mice and *Ankrd55^fl/fl^* *LCK^Cre^* mice. (**B**) Mean clinical score of EAE in *Ankrd55^fl/fl^* mice and *Ankrd55^fl/fl^* *LCK^Cre^* mice (*n* = 9 or 10 mice per group) induced by active immunization with MOG_35–55_. (**C** and **D**) Flow cytometry analysis of infiltrated immune cell in the brain at the peak of EAE disease in *Ankrd55^fl/fl^* mice and *Ankrd55^fl/fl^* *LCK^Cre^* mice, including CD4^+^ T cells, CD8^+^ T cells, B cells, neutrophils, and monocytes. Data are presented as a summary plot of absolute cell counts (**C**) and a representative plot (**D**). (**E**) RT-qPCR analysis of inflammatory gene expression in the spinal cord during peak disease in *Ankrd55^fl/fl^* mice and *Ankrd55^fl/fl^ LCK^Cre^* mice. (**F**) Representative histological images of spinal cord sections from *Ankrd55^fl/fl^* and *Ankrd55^fl/fl^* *Lck^Cre^* mice at peak disease. LFB, Luxol fast blue. (**G**) Representative immunofluorescence staining of spinal cord sections from *Ankrd55^fl/fl^* and *Ankrd55^fl/fl^* *Lck^Cre^* mice at the peak of EAE. Sections were stained with antibodies against CD4, Ki67, and with DAPI. For quantification, 3 fields with evident immune cell infiltration were captured per mouse, and the percentage of Ki67^+^ cells among CD4^+^ T cells was calculated (*n* = 4 mice per group). Scale bars: 5 μm. (**H**) Schematic representation of the experiments in **I** and **J**. (**I** and **J**) Mean clinical score of EAE mice (*n* = 9 or 10 mice per group) induced by adoptive transfer of MOG-reactive Th1 (**I**) and Th17 (**J**) cells. *P < 0.05, **P < 0.01, ***P < 0.001, ****P < 0.0001, based on unpaired, 2-tailed *t* test (**C**, **E**, and **G**) or 2-way ANOVA with Tukey’s multiple-comparison test (**B**, **I**, and **I**). Data are shown as mean ± SEM.

Based on our findings highlighting the critical role of ANKRD55 in CD4^+^ T cell biology, we then investigated whether targeted depletion of *Ankrd55* in T cells would mitigate EAE development. We generated T cell–specific *Ankrd55*^–/–^ mice by crossing *Ankrd55^fl/fl^* mice with *Lck^Cre^* mice (Figure 4A and Supplemental Figure 4). Compared with *Ankrd55^fl/fl^* control mice, T cell–conditioned *Ankrd55*^–/–^ mice exhibited significantly reduced disease severity after MOG_35–55_ immunization (Figure 4B). Importantly, we observed a marked decrease in immune cell infiltration into the brain at the peak of EAE, including reductions in CD4^+^ T cells, CD8^+^ T cells, B cells, monocytes, and neutrophils (Figure 4, C and D), along with diminished expression levels of inflammatory factors and chemokines in the spinal cord (Figure 4E). Additionally, we analyzed Th1, Th17, and Treg populations within the CNS at the peak of EAE (Supplemental Figure 4, B and C). Our results show that, compared with *Ankrd55^fl/fl^* mice, *Ankrd55^fl/fl^* *Lck^Cre^* mice exhibited a significant reduction in both Th1 and Th17 cell infiltration in the CNS, while the proportion of Treg cells remained unchanged. To exam in situ proliferation of CD4^+^ T cells within the EAE spinal cord, we performed Ki67 staining in combination with CD4 in CNS (Figure 4G). We found that deletion of *Ankrd55* impaired the proliferation of CD4^+^ T cells. Histopathological analysis further revealed reduced immune cell accumulation and associated demyelination in the spinal cords of T cell–specific *Ankrd55*^–/–^ mice compared with control mice (Figure 4F).

To evaluate the impact of *Ankrd55* deficiency on T cell function during EAE, we isolated splenocytes from WT and *Ankrd55*^–/–^ mice and polarized them to Th1 or Th17 cells. These Th1 or Th17 cells were adoptively transferred to irradiated WT recipients for EAE induction (Figure 4H). Our results indicated that mice receiving *Ankrd55*^–/–^ cells exhibited significantly reduced clinical scores compared with those receiving WT cells, regardless of whether the transferred cells were Th1 or Th17 subsets (Figure 4, I and J, and Supplemental Figure 5A). These findings collectively suggest that T cell–intrinsic ANKRD55 is essential for both active EAE and adaptive EAE progression.

### ANKRD55 promotes TCR signal transduction.

**Figure 5:**
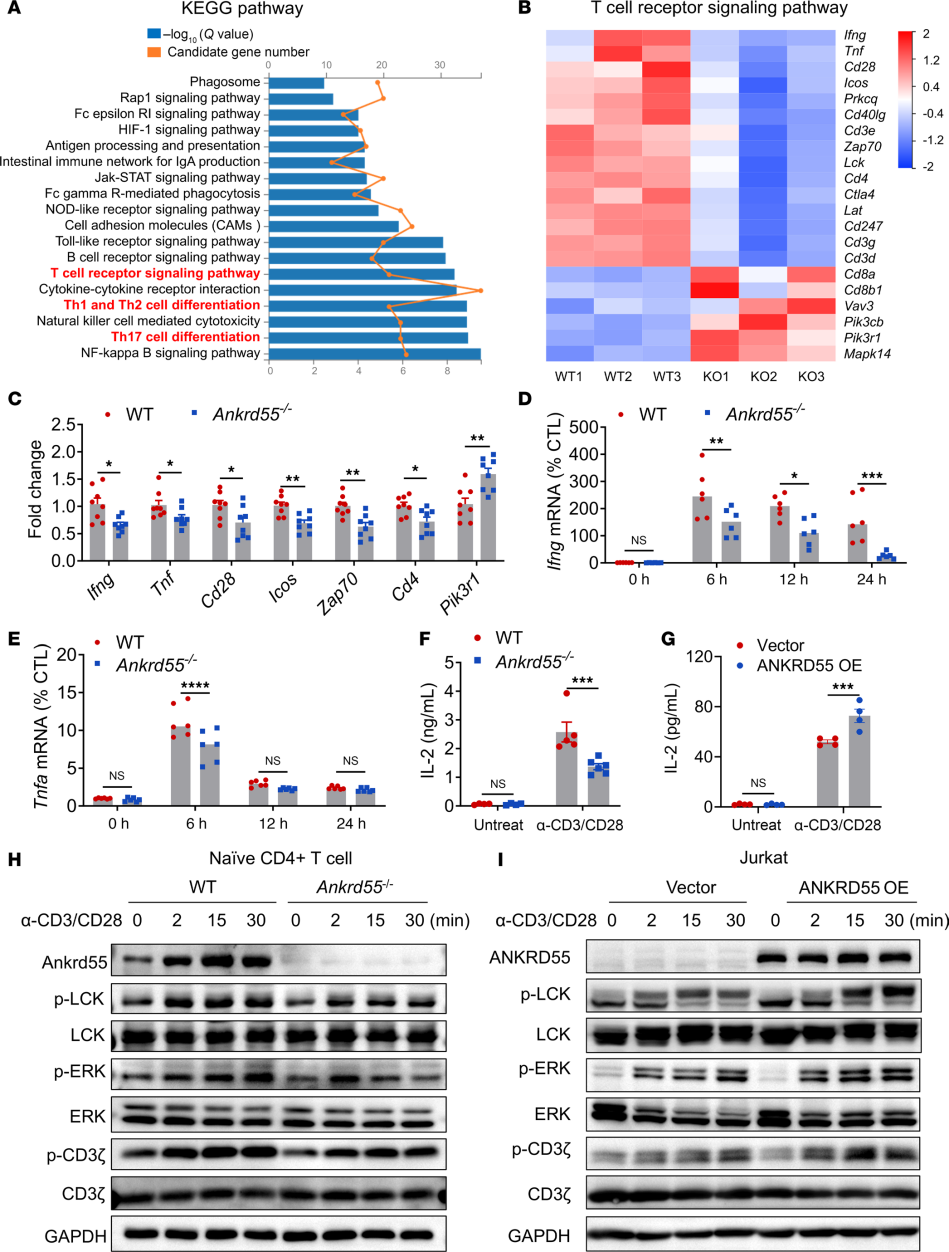
ANKRD55 promotes TCR signal transduction. (**A**) Through KEGG pathway analysis, the most significantly enriched signaling pathway in CD4^+^ T cells isolated from the spleens of WT and *Ankrd55*^–/–^ mice was identified on day 10 after EAE induction. (**B**) Heatmap analysis of the TCR signaling pathway revealed genes with adjusted *P* value < 0.05, FDR < 0.05, and log_2_(fold change) > 1.2 in RNA-Seq data from CD4^+^ T cells of 3 pairs of WT and *Ankrd55*^–/–^ mice on day 10 after EAE induction. (**C**–**E**) RT-qPCR analysis of CD4^+^ T cells isolated from the spleens of WT and *Ankrd55*^–/–^ mice on day 10 after EAE induction (*n* = 8) demonstrated differential expression of target genes. (**F**) CD4^+^ T cells were isolated from the spleens of WT and *Ankrd55*^–/–^ mice and cultured on plates coated with anti-CD3 and anti-CD28 for 48 hours. IL-2 secretion levels were analyzed using ELISA. (**G**) Measurement of IL-2 secretion in ANKRD55-overexpressing Jurkat cells. Jurkat cells stably overexpressing ANKRD55 or transfected with empty vector were cultured on plates coated with anti-CD3 and anti-CD28 for 48 hours. Culture supernatants were collected, and IL-2 levels were quantified by ELISA. (**H** and **I**) Immunoblot analysis of TCR signaling in primary and transformed T cells. (**H**) Naive CD4^+^ T cells isolated from WT and *Ankrd55*^–/–^ mice were stimulated on plates coated with anti-CD3 and anti-CD28 for 0, 2, 15, or 30 minutes. Cell lysates were collected and subjected to immunoblotting to assess activation of TCR signaling pathways. (**I**) Jurkat cells stably overexpressing ANKRD55 or transfected with empty vector were treated under the same stimulation conditions, and protein lysates were analyzed by immunoblotting. *P < 0.05, **P < 0.01, ***P < 0.001, ****P < 0.0001, based on unpaired, 2-tailed *t* test (**C**–**G**). Data are shown as mean ± SEM.

Given the critical role of ANKRD55 in CD4^+^ T cells, we further investigated the molecular mechanisms by which ANKRD55 mediates its effects. CD4^+^ T cells were sorted from mice spleens 10 days after MOG_35–55_ immunization and analyzed by RNA-Seq. Kyoto Encyclopedia of Genes and Genomes (KEGG) analysis indicated that the top pathways downregulated in *Ankrd55*^–/–^ CD4^+^ T cells primarily included the TCR signaling pathway, Th1 and Th17 cell differentiation, and other processes (Figure 5A). Consistently, our previous results of single-cell analysis also suggested an important role of ANKRD55 in CD4^+^ T cell differentiation (Figure 2, M and N). Taken together, these results support the hypothesis that ANKRD55 plays a role in the early stages of T cell activation. Since the TCR is indispensable for the differentiation and activation of T cell subsets, we subsequently analyzed the TCR signaling pathway and found that expression of several TCR-associated molecules (such as Zap70, Pik3r1, CD3, LCK, and others) was significantly affected in *Ankrd55*^–/–^ mice (Figure 5B). To confirm these results, CD4^+^ T cells were isolated from the spleen of EAE mice followed by RT-qPCR analysis, revealing a similar pattern of expression (Figure 5C). Among the TCR-related genes, 2 particularly important ones encoded the proinflammatory cytokines IFN-γ and TNF-α, which are secreted by activated inflammatory T cells. To evaluate the expression of IFN-γ and TNF-α, we isolated CD4^+^ T cells from naive *Ankrd55*^–/–^ or WT mice, stimulated them with antibodies against CD3 and CD28 for up to 24 hours, and performed the RT-qPCR analysis. Under baseline conditions, there was no discernible difference in the expression of *Ifng* and *Tnfa* between the 2 groups. Following a 6-, 12-, and 24-hour stimulation with antibodies, CD4^+^ T cells from *Ankrd55*^–/–^ mice exhibited significantly reduced expression of *Ifng* compared with WT mice (Figure 5D). Similarly, CD4^+^ T cells from *Ankrd55*^–/–^ mice showed markedly diminished *Tnfa* expression compared with WT mice 6 hours after antibody stimulation (Figure 5E). Secretion of IL-2, a cytokine associated with T cell proliferation and activation, was examined in CD4^+^ T cells isolated from *Ankrd55*-KO and WT mice 48 hours after stimulation with antibodies against CD3 and CD28. The results demonstrated that *Ankrd55* deficiency in CD4^+^ T cells inhibited IL-2 secretion, providing additional evidence for a role of ANKRD55 in TCR signaling (Figure 5F). Consistently, *Ankrd55* deficiency was associated with reduced TCR signaling activation, which was revealed by detecting phosphorylation of CD3ζ, LCK, and ERK in naive CD4^+^ T cells (Figure 5H and Supplemental Figure 8C). In line with the data obtained from primary CD4^+^ T cells, overexpression of ANKRD55 in Jurkat cells, an immortalized human T lymphocyte cell line, led to a marked enhancement of IL-2 production and activation of the TCR signaling pathway (Figure 5, G and I, and Supplemental Figure 8D). Together, these findings suggest that ANKRD55 plays a key role in promoting the activation of the TCR signaling pathway.

### ANKRD55 interacts with multiple subunits of the CCT complex required for TCR activation.

**Figure 6:**
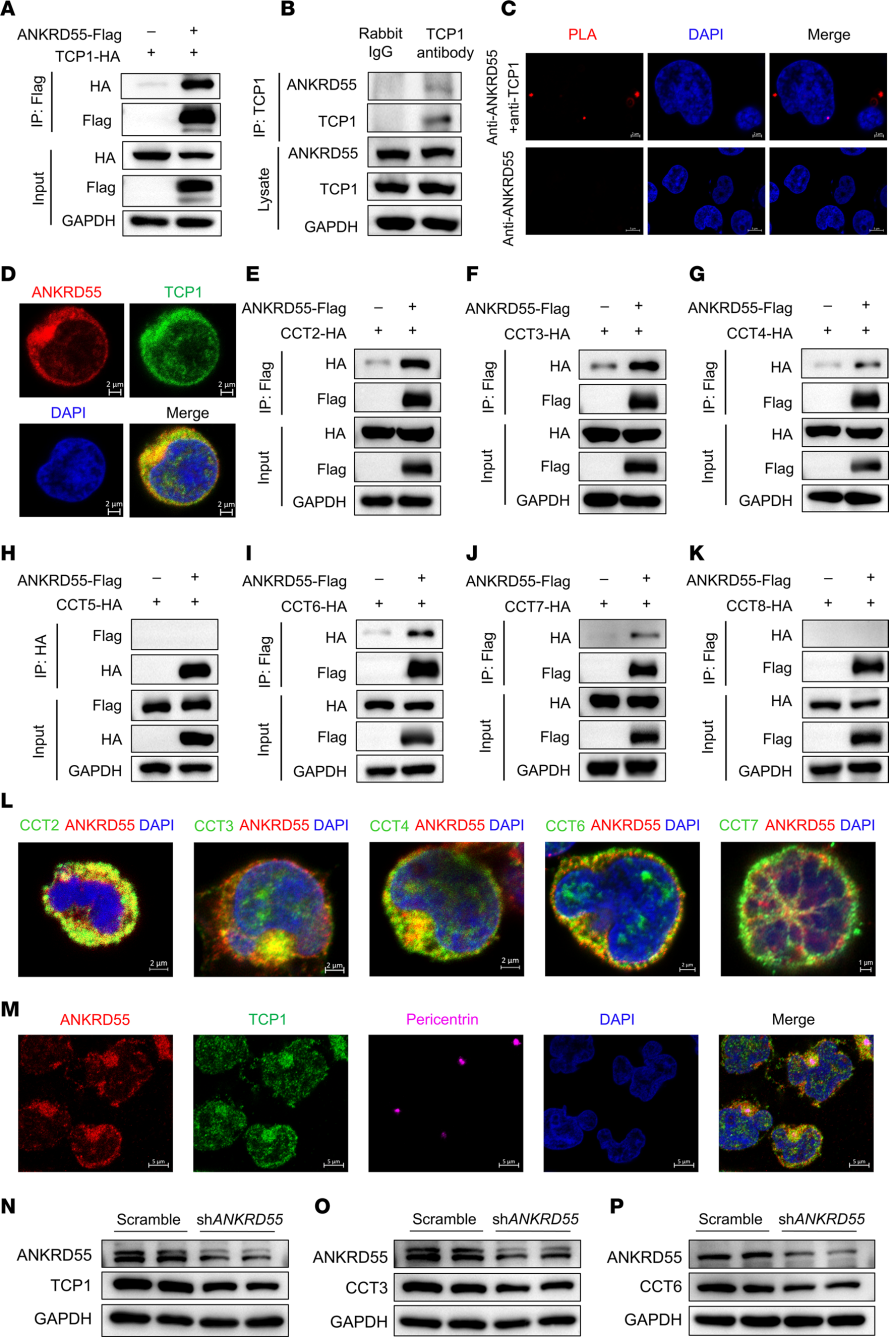
ANKRD55 interacts with multiple subunits of the CCT complex required for TCR activation. (**A**) Overexpression of ANKRD55 and TCP1 in HEK293T cells followed by co-IP analysis to determine their interaction. (**B**) Protein extraction from Jurkat cells with subsequent co-IP assay to determine the interaction between ANKRD55 and TCP1. (**C**) PLA experiment in Jurkat cells to assess the interaction between ANKRD55 and TCP1. Cells were fixed and incubated with primary antibodies against ANKRD55 and TCP1, followed by PLA probe ligation and amplification. Red fluorescent puncta indicate close proximity (<40 nm) between ANKRD55 and TCP1, suggesting a direct or complex-mediated interaction. Nuclei were counterstained with DAPI (blue). Representative images are shown. Scale bars: 2 μm (top), 5 μm (bottom). (**D**) Immunofluorescent staining of Jurkat cells to analyze the colocalization of ANKRD55 and TCP1. Scale bars: 2 μm. (**E**–**K**) Co-IP assays were performed using HEK293T cells to investigate the interactions between ANKRD55 and individual CCT subunits, including CCT2, CCT3, CCT4, CCT5, CCT6, CCT7, and CCT8. (**L**) Immunofluorescent staining of Jurkat cells to assess colocalization between ANKRD55 and specific CCT subunits (CCT2, CCT3, CCT4, and CCT7). Scale bars: 2 μm (first 4 panels), 1 μm (fifth panel). (**M**) Immunofluorescent staining of Jurkat cells to visualize the subcellular localization of ANKRD55, TCP1, and pericentrin. Scale bars: 5 μm. (**N**–**P**) Immunoblotting analysis of TCP1 (**N**), CCT3 (**O**), and CCT6 (**P**) expression levels in Jurkat cells following *ANKRD55* knockdown.

Although ANKRD55 is tightly associated with TCR activation and T cell inflammation, the molecular mechanism of ANKRD55 function remains unknown. Given that the ankyrin repeat domain is one of the most prevalent protein-protein interaction motifs, ANKRD55 is likely involved in forming complexes with other proteins. To identify ANKRD55 interactors, we generated a stable Jurkat cell line overexpressing ANKRD55 and performed co-IP followed by mass spectrometry. We selected 10 proteins that have the potential to bind to ANKRD55 and verified whether they bind to ANKRD55 by co-IP experiments. T-complex protein 1 (TCP1) was identified as an interacting protein of ANKRD55 (Figure 6A). TCP1 is one of the subunits of TCP1-ring complex/CCT, also called CCT1. Previous studies have shown that the CCT controls changes in centromere mutual orientation and polarization of tubulin dynamics induced by TCRs in immune synapse–forming T lymphocytes, by which CCT controls T cell activation and polarity (33). This is consistent with our findings regarding the function of ANKRD55 in CD4^+^ T cells. We next demonstrated the endogenous interaction of ANKRD55 with TCP1 by co-IP assay in Jurkat cells (Figure 6B and Supplemental Figure 6A). We further validated the interaction between ANKRD55 and TCP1 by the proximity ligation assay (PLA), a highly sensitive technique that enables in situ detection of protein-protein interactions by generating a fluorescent signal only when 2 target proteins are in close proximity (Figure 6C), and immunofluorescence imaging (Figure 6D). Next, we engineered ANKRD55 and TCP1 fragments based on structural domains and explored which domains are involved in the interaction of ANKRD55 with TCP1 by co-IP experiments (Supplemental Figure 6, B and C), revealing that ANKRD55 (324–453 aa) and TCP1 (1–187 aa) are essential regions for their interaction. CCT is the most complex molecular chaperone, consisting of 8 parallel homologous subunits (CCT1–CCT8) assembled in a fixed order to form a double-ring structure, with the 2 rings stacked to form a barrel-shaped chamber and each ring containing 8 different subunits (34). We found that the mass spectrometry results of co-IP also contained other subunits of CCT, which suggested that ANKRD55 may function by interacting with TCP1 and its associated CCT complex. Therefore, we constructed plasmids expressing the other 7 subunits of the CCT complex to study their interactions with ANKRD55 by co-IP. The results indicated that ANKRD55 interacted with all subunits except CCT5 and CCT8 (Figure 6, E–K). These findings were verified by immunofluorescence imaging for protein colocalization (Figure 6L). We found that subunits of the CCT complex and ANKRD55 often colocalized significantly in a region near the nucleus. The centrosomes were always located in the cytoplasm near the nucleus, close to the center of the cell. Given the location where colocalization occurs, we hypothesized that ANKRD55 and CCT might aggregate near the centrosome. This was confirmed by immunofluorescence imaging, which revealed that ANKRD55 and TCP1 not only aggregated near the centrosomes, but also colocalized in the centrosomes (Figure 6M). Furthermore, knockdown of *ANKRD55* in Jurkat cells led to a reduction in the expression of multiple subunits of the CCT complex, suggesting ANKRD55 is essential for CCT complex formation and stability (Figure 6, N–P, Supplemental Figure 6, D–H, and Supplemental Figure 8E). In summary, we have demonstrated the interaction of ANKRD55 with multiple subunits of CCT using orthogonal experimental methods. Our data suggest that ANKRD55 plays a significant role in the assembly and function of the CCT complex.

### ANKRD55 affects CCT complex assembly by competing with CCT5 binding to CCT1/3/6, thus promoting immune synapse formation and TCR activation.

**Figure 7:**
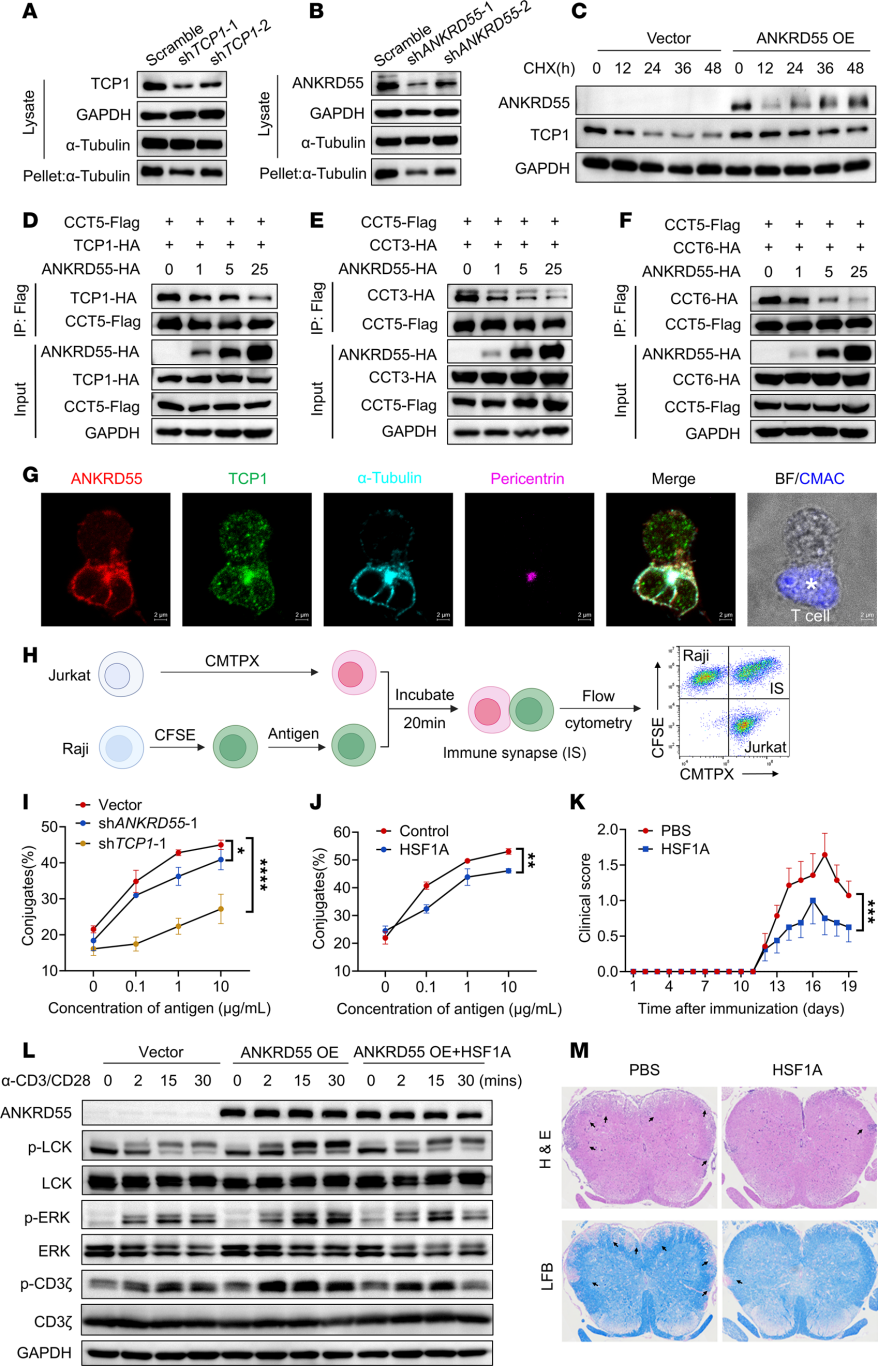
ANKRD55 affects CCT complex assembly by competing with CCT5 binding to CCT1/3/6, thus promoting immune synapse formation and TCR activation. (**A** and **B**) α-Tubulin immunoblotting in lysates and pellet determined by microtubule sedimentation assay in Jurkat cells with *TCP1* (**A**) or *ANKRD55* (**B**) knocked down. (**C**) TCP1 degradation rate analyzed via immunoblot after cycloheximide (CHX; 70 μM) treatment for 0–48 hours in control and Jurkat cells overexpressing ANKRD55. (**D**–**F**) Co-IP detection of interactions between CCT5 and TCP1 (**D**), CCT3 (**E**), or CCT6 (**F**) at varying concentrations of ANKRD55 in HEK293T. (**G**) Immunofluorescence analysis of immune synapse formation between Jurkat and Raji cells. Jurkat cells were prelabeled with CMAC. Raji cells were stimulated with SEE for 30 minutes. The 2 cell types were then cocultured for 30 minutes. Cells were stained with antibodies against ANKRD55, TCP1, pericentrin, and α-tubulin to visualize protein localization at the immune synapse. Scale bars: 2 μm. BF, bright-field; CMAC, CellTracker blue fluorescent probe. (**H**) Flow cytometry–based immune synapse (IS) pattern analysis. (**I** and **J**) Raji cells (APCs) stained with CFSE and stimulated with SEE for 30 minutes at 37°C and Jurkat cells (T cells) stained with CMTPX. T cell conjugation with APCs after 20 minutes of contact was analyzed by flow cytometry. Conjugate percentages were determined for Jurkat cells with *ANKRD55* or *TCP1* knocked down (**I**) and pretreatment with HSF1A (50 μM) for 2 hours (**J**). (**K**) Mean clinical score of EAE in mice injected intraperitoneally with PBS or HSF1A (20 mg/mL) (*n* = 7 or 8 mice per group), induced by active immunization with MOG_35–55_. (**L**) Immunoblot analysis of TCR signaling in Jurkat cells. Cells included vector control, a stable ANKRD55-overexpressing cell line, and ANKRD55-overexpressing cells pretreated with HSF1A (50 μM, 2 hours). All groups were stimulated on plates coated with anti-CD3 and anti-CD28. Lysates were collected at the indicated time points (1, 2, 15, and 30 minutes) and probed for TCR signaling–associated proteins. (**M**) H&E and Luxol fast blue (LFB) staining of spinal cord sections at the peak of EAE disease. Arrows indicate areas of demyelination. *P < 0.05, **P < 0.01, ***P < 0.001, ****P < 0.0001, by 2-way ANOVA with Tukey’s multiple-comparison test. Data are shown as mean ± SEM.

The CCT complex is essential for proper folding of actin and tubulin. To determine the role of ANKRD55 in CCT complex assembly, we conducted an in vitro microtubule sedimentation assay. Our results showed that α-tubulin successfully precipitated under polymerization conditions in control Jurkat cells, while *TCP1* knockdown significantly reduced the levels of precipitated α-tubulin, confirming the function of TCP1 in α-tubulin polymerization (Figure 7A and Supplemental Figure 9A). Consistently, *Ankrd55* knockdown significantly impaired microtubule polymerization compared with controls (Figure 7B and Supplemental Figure 9A). Overexpression of ANKRD55 in Jurkat cells treated with cycloheximide, an inhibitor of eukaryotic translation, prevented the degradation of TCP1, indicating that ANKRD55 stabilizes CCT subunits (Figure 7C and Supplemental Figure 9B). The assembly of CCT subunits is a tightly regulated process, often ending in sequential and precise CCT protein interactions. CCT5 has been identified as a key player in regulating CCT assembly, whereas misassembled CCT subunits are typically marked for degradation (35). Through co-IP experiments, we observed that increasing ANKRD55 levels reduced the interaction between CCT5 and CCT1/3/6 (Figure 7, D–F, Supplemental Figure 7, C–E, and Supplemental Figure 9C). Previous studies have highlighted the role of TCP1, CCT3, and CCT6 in the final stages of CCT assembly (35). Based on these findings, we hypothesize that ANKRD55 may compete with CCT5 for binding to TCP1, CCT3, and CCT6, thereby preventing misassembly and degradation.

The microtubule-organizing center (MTOC) plays a crucial role in immune synapse formation. The MTOC of animal cells is the centrosome. During T cell activation, the MTOC translocates to the site of contact with APCs (36). Our colocalization studies revealed an interplay between ANKRD55 and TCP1 at the MTOC during immune synapse formation (Figure 7G). Given that CCT is known to assist in immune synapse formation by modulating tubulin folding (33), we propose that ANKRD55 may interact with CCT to facilitate immune synapse formation and enhance TCR signaling.

To validate these hypotheses, a human B cell line Raji was labeled with CFSE and stimulated with the staphylococcal enterotoxin E (SEE) antigen, followed by incubation with Jurkat cells labeled with CMTPX (CellTracker red fluorescent probe). Our data show that knockdown of either *TCP1* or *ANKRD55* significantly inhibited immune synapse formation, as detected by flow cytometry (Figure 7, H and I). Consistently, overexpression of ANKRD55 promoted synapse formation (Supplemental Figure 7F). Furthermore, treatment with HSF1A, a TCP1 inhibitor, reduced both the immune synapse formation and the EAE severity in animal models (Figure 7, J–M, Supplemental Figure 7, H and I, and Supplemental Figure 9D). Together, these findings demonstrate that ANKRD55 facilitates proper CCT complex assembly and microtubule polymerization, thereby promoting immune synapse formation and T cell activation through stabilization of CCT subunits and modulation of tubulin dynamics.

## Discussion

MS is a classic T cell–driven autoimmune inflammatory disease characterized by pathogenic T cell activation and CNS demyelination. However, the precise mechanisms for specifically targeting these inflammatory T cells remain unclear. In our current study, we identified that *ANKRD55* expression was significantly upregulated in both PBMCs and cells of the CSF isolated from MS patients, as well as in the spleens and lymph nodes of EAE mice. Our analysis revealed that among various peripheral immune cells, *ANKRD55* expression is predominantly present in CD4^+^ T cells. Data from single-cell sequencing obtained from the GEO database corroborated this observation. Furthermore, we discovered that *ANKRD55* exhibited higher expression levels in naive CD4^+^ T cells, suggesting a potential role for ANKRD55 in the early stages of T cell differentiation. Chu et al. similarly identified *ANKRD55* in a single-cell analysis of 308,048 transcriptomes of T cells from 16 cancer types as a marker for naive CD4^+^ T subpopulations (37). Notably, both Th1 and Th17 subsets are considered key pathogenic cell types in EAE models. Our study demonstrated that the absence of *Ankrd55* impaired the conversion of naive CD4^+^ T cells into Th1 and Th17 cells. Additionally, genetic deletion or cell-specific deletion of *Ankrd55* led to a significant reduction in disease severity and neuroinflammation in the EAE model. Moreover, transfer of *Ankrd55*^–/–^ pathogenic Th1 or Th17 cells into WT mice also attenuated disease progression, indicating that *Ankrd55* deficiency not only inhibits the differentiation of Th1 and Th17 cells but also reduces their pathogenicity. Collectively, these findings underscore the critical role that ANKRD55 plays in the T cell–driven inflammation observed in MS.

Our RNA-Seq analysis revealed a significant downregulation of TCR signaling pathways in CD4^+^ T cells lacking *Ankrd55* expression. This discovery was further supported by our experimental findings, which demonstrated that *Ankrd55* deficiency perturbs key events within the TCR signaling cascade, particularly affecting phosphorylation of downstream molecules, such as CD3ζ and LCK. Notably, these phosphorylation events occur at the initial stages of the signaling pathway, suggesting that ANKRD55 may play a critical regulatory role upstream of T cell activation.

T cell activation is initiated by contact with APCs that present antigenic MHC-peptide complexes. T cells become activated upon the formation of stable T cell–APC junctions, commonly referred to as immune synapses. As a component of these immune synapses, the T cell cytoskeleton undergoes reorganization to provide structural support for the formation and stabilization of immune synapses (38). Our studies reveal that ANKRD55 interacts with several subunits of the CCT complex, which functions as a molecular chaperone regulating microtubule-associated proteins. Furthermore, we observed that ANKRD55 aggregates near centrosomes in association with TCP1 under normal cellular conditions. These findings align with observations from Martin-Cofreces et al., who demonstrated that the CCT complex is responsible for modulating the mutual orientation of centromeres and the polarization of tubulin dynamics induced by TCR activation during immune synapse formation in T lymphocytes (33). These discoveries collectively suggest that ANKRD55 and TCP1 may together regulate tubulin dynamics, a process critical for immune synapse formation and function.

Molecular CCT accumulates in the activated centrosome, where it serves as a dynamic hub for the folding and quality control of newly synthesized proteins (33). CCT plays a pivotal role in the folding of several key proteins, including actin, α-tubulin, and β-tubulin (34, 39). Specifically, CCT promotes the formation of α- and β-tubulin heterodimers that subsequently incorporate into nascent microtubules with the aid of tubulin-binding cofactors. Our findings suggest that ANKRD55 is essential for microtubule protein polymerization, as its knockdown disrupts CCT assembly and impairs proper tubulin polymerization. CCT is a 1 MDa hetero-oligomeric complex composed of 2 rings made up of 8 distinct 60 kDa subunits (40). Betancourt Moreira et al. indicated that the CCT subunits must assemble in a specific, hierarchical manner, and improper assembly can lead to degradation (35). Among these subunits, CCT5 is particularly prone to misassembly with other subunits, resulting in the degradation of multiple CCT components (35). Our research demonstrates that ANKRD55 competes with CCT5 for binding to TCP1, CCT3, and CCT6 during the assembly process. Specifically, TCP1, CCT3, and CCT6 are incorporated into the complex at the final stage of assembly, and their interaction with CCT5 leads to its degradation. This suggests that ANKRD55 may play a critical role in regulating CCT assembly by preventing the interaction between CCT5 and these subunits. However, further investigation is required to fully elucidate the mechanism of CCT assembly.

It has been known that single nucleotide polymorphisms in *ANKRD55* are increasingly recognized as contributors to a variety of autoimmune disorders, including MS, rheumatoid arthritis, and type 2 diabetes (30, 41–44). We corroborated these findings by demonstrating that carriers of the rs6859219C/C allele exhibit elevated proportions of Th1 and Th17 cells as well as GM-CSF^+^CD4^+^ T cells compared with noncarriers (rs6859219C/A). These observations may provide insights into the mechanisms underlying the roles of ANKRD55 and T cell inflammation in autoimmune conditions.

In conclusion, our study highlights a critical regulatory role of ANKRD55 in immune synapse formation and TCR signaling through its involvement in CCT assembly. These findings also provide a mechanistic basis for the genetic association of *ANKRD55* with MS, offering new avenues for therapeutic development. By uncovering what we believe to be a previously unrecognized link between ANKRD55 and T cell inflammation, this study advances our understanding of autoimmune pathogenesis and lays the groundwork for targeted interventions. While the role of ANKRD55 in other autoimmune conditions remains a topic for further investigation, targeting ANKRD55 and its associated pathways presents promising therapeutic opportunities. Interventions designed to modulate ANKRD55 activity could potentially restore the immune synapse balance and mitigate aberrant T cell activation. Small-molecule inhibitors of the ANKRD55-CCT interaction or agents regulating tubulin polymerization could serve as strategies to dampen inflammatory responses in autoimmune conditions.

## Methods

### Sex as a biological variable.

Our study examined male and female animals, and similar findings are reported for both sexes.

### Mice.

The *Ankrd55*-KO mice were generated by GemPharmatech. The *Ankrd55* gene has 6 transcripts. Exon 3–exon 4 of the *Ankrd55*-201 (ENSMUST00000022275.13) transcript, containing 241 bp coding sequence, was depleted by the CRISPR/Cas9 technique. *Lck^Cre^* and *Ankrd55^fl/fl^* mice (strain S-CKO-16666) were purchased from Cyagen. Exon 3 was selected as a conditional knockout region. The region contains 131 bp coding sequence. To engineer the targeting vector, homologous arms and the conditional knockout (cKO) region were generated by PCR using BAC clone RP23-284J12 as template. Ribonucleoprotein and targeting vector were coinjected into fertilized eggs for cKO mouse production. The knockout of exon 3 resulted in frameshift of the gene and covered 6.98% of the coding region.

The size of intron 2 for 5′-loxP site insertion was 4,504 bp, and the size of intron 3 for 3′-loxP site insertion was 3,946 bp. The size of the effective cKO region was approximately 1.1 kb. Mice were kept in a specific pathogen–free environment.

### Cells.

HEK293T cells were maintained in DMEM plus 10% FBS and 1% penicillin-streptomycin. Jurkat cells and Raji cells were maintained in RPMI 1640 plus 10% FBS, 1% penicillin-streptomycin, and 1% sodium pyruvate. The HEK293T, Jurkat, and Raji cell lines were purchased from the Cell Bank of the Chinese Academy of Sciences (Shanghai, China).

### Reagents.

Phospho-CD3ζ (Tyr142) (catalog 67748) antibody was purchased from Cell Signaling Technology. Anti-pericentrin (catalog ab28144) and anti-Ki67 (catalog ab15580) antibodies were purchased from Abcam. Anti-ANKRD55 antibodies (catalog HPA051049 and HPA061649) were purchased from Sigma-Aldrich. α-Tubulin monoclonal antibody (catalog MA1-80017), CD4 monoclonal antibody (catalog MA1146), and the mouse and human IL-2 uncoated ELISA kits were purchased from Invitrogen. TCP1 antibody (catalog 10320 and 68183), CCT2 antibody (catalog 68214), CCT3 antibody (catalog 60264), CCT4 antibody (catalog 67455), CCT5 antibody (catalog 67400), CCT6 antibody (catalog 19793), CCT7 antibody (catalog 68214), DYKDDDDK tag polyclonal antibody (catalog 20543), HA tag polyclonal antibody (catalog 51064), phospho-ERK1/2 (Thr202/Tyr204) polyclonal antibody (catalog 28733), CD247 polyclonal antibody (catalog 12837), and phospho-LCK-Y394 rabbit antibody (catalog AP0182) were purchased from Proteintech. PerCP/Cyanine5.5 anti-mouse CD45 (catalog 103132), Brilliant Violet 650 anti-mouse CD3 (catalog 100229), Alexa Fluor 488 anti-mouse CD4 (catalog 100423), Brilliant Violet 421 anti-mouse CD25 (catalog 102034), Brilliant Violet 421 anti-mouse IL-17A (catalog 506926), PE anti-mouse IFN-γ (catalog 505808), FITC anti–T-bet (catalog 644811), PE anti-mouse IL-13 (catalog 159403), APC anti-mouse CD69 (catalog 104514), APC anti-human GM-CSF (catalog 502310), Brilliant Violet 421 anti-mouse Ly-6G (catalog 127628), Brilliant Violet 605 anti-mouse Ly-6C (catalog 128036), APC anti-mouse/human CD11b (catalog 101212), PE anti-mouse F4/80 (catalog 123110), APC anti-mouse CD69 (catalog 104514), and TruStain FcX (anti-mouse CD16/32) (catalog 101319) were purchased from BioLegend. Fixable Viability Dye eFluor 780 and PE FOXP3 monoclonal antibody (FJK-16s) (catalog 12577382) were purchased from eBioscience. PE mouse anti-mouse RORγt (catalog 562607), BV510 mouse anti-human CD45 (catalog 563204), BB515 mouse anti-human CD4 (catalog 564500), BV650 mouse anti-human IL-17A (catalog 563746), and PE-Cy7 mouse anti-human IFN-γ (catalog 557643) were purchased from BD Pharmingen.

### Single-cell RNA-Seq data analysis.

Single-cell transcriptomic data from CSF cells and PBMCs of MS patients and healthy controls were obtained from the GEO database (GSE138266), as published by Schafflick et al. (29). The data were analyzed using Seurat (v4.4.0) in R. Cells with more than 100 and fewer than 6,000 RNA features were selected for further analysis. Cells with greater than 20% mitochondrial genes were also removed. Genes detected in fewer than 3 cells were excluded. Integration across samples was achieved using the top 1,000 features with the highest dispersion for each dataset using the following Seurat commands: SelectIntegrationFeatures, PrepSCTIntegration, FindIntegrationAnchors, and IntegrateData. CD4^+^ T cells were identified based on canonical marker gene expression and further stratified into naive and effector subpopulations using unsupervised clustering and known marker profiles. Differential expression analysis was conducted to compare *ANKRD55* expression between MS patients and healthy controls. Pseudotime trajectory analysis was performed using Monocle 2 to examine the dynamic expression pattern of *ANKRD55* during CD4^+^ T cell differentiation.

### RNAi.

shRNAs were obtained from Tsingke and target sequences as follows: *ANKRD55* shRNA-1, GCCCTTGATGCATGCGGTTTC; *ANKRD55* shRNA-2, GCGGGCTTCAGCGATATTATT; *TCP1* shRNA-1, GCAAGATCACTTCTTGTTATT; and *TCP1* shRNA-2, GCTCTTTACATGATGCACTTT.

### Immunoblot and immunoprecipitation.

Cells were lysed by RIPA buffer (Beyotime P0013B) with protease inhibitors (MedChemExpress HY-K0010), phosphatase inhibitors (MedChemExpress HY-K0021), and PMSF (Beyotime ST506). Cell or tissue samples were lysed with precooled RIPA buffer and sonicated at 60 Hz for 2 minutes. Clarified supernatant was collected after centrifugation at 16,000*g* for 10 minutes. Then, 5× loading buffer was added according to the volume of protein samples, and the proteins were denatured by boiling in a metal bath at 100°C for 5 minutes. Fifty micrograms of protein lysate per lane was run on a 10% or 7.5% SDS-PAGE gel and immunoblotted with different antibodies. For the immunoprecipitation assay, cell lysates were incubated with Protein A/G Magnetic Beads (MedChemExpress HY-K0202) coupled to 1 μg of antibody overnight at 4°C, beads were washed 4 times with lysis buffer, and precipitates were eluted with 1× sample buffer. The eluate and whole-cell extracts were separated on SDS-PAGE and then immunoblotted with antibodies. Grayscale quantification of protein blots was performed on images of scanned films using ImageJ (NIH) software.

### RNA isolation and RT-qPCR.

Total RNA was extracted with a FastPure Cell/Tissue Total RNA Isolation Kit V2 (Vazyme) and reverse transcribed to cDNA, and the genomic DNA was removed with HiScript II Q RT SuperMix for qPCR (Vazyme). cDNA was subsequently used for RT-qPCR with Genious 2× SYBR Green Fast qPCR Mix (Vazyme) on a LightCycler 96 system (Roche Diagnostics) according to the manufacturer’s instructions. The cDNA was then used for RT-qPCR with AceQ Universal SYBR qPCR Master Mix (Vazyme) on a LightCycler 96 system according to the manufacturer’s instructions. RT-qPCR data were analyzed by calculating the change in relative gene expression levels using the comparative ΔΔCt method.

### CD4^+^ T cell differentiation.

Naive CD4^+^ T cells were obtained by magnetic bead sorting using the Naive CD4^+^ T cell isolation kit (Miltenyi). For Th1 differentiation, 20 ng/mL IL-12 (R&D Systems) and 5 μg/mL anti–IL-4 (BioLegend) were added to the medium. For Th17 differentiation 10 ng/mL TGF-β (R&D Systems), 20 ng/mL IL-6 (R&D Systems), 5 μg/mL anti–IL-4, 5 μg/mL anti–IFN-γ (BioLegend), 10 ng/mL IL-1β (R&D Systems), and 20 ng/mL IL-23 (R&D Systems) were added. For Th2 differentiation, 20 ng/mL IL-4 (R&D Systems) and 10 μg/mL anti–IFN-γ were added. For Treg differentiation, 10 ng/mL TGF-β, 5 μg/mL anti–IL-4, and 5 μg/mL anti–IFN-γ were added to the medium. These cells were all cultured for 4 days on plates encapsulated in anti-CD3/CD28 (1 μg/mL).

### CD4^+^ T cell priming with MOG_35–55_.

Spleens from mice on day 10 after EAE-induced immunization were taken and isolated as single cells and cultured with 25 μg/mL MOG. For Th1 polarization, 20 ng/mL IL-12 was added; for Th17 polarization, 10 ng/mL IL-1β and 20 ng/mL IL-23 were added. The cells were cultured for 3–5 days.

### Induction and assessment of active EAE.

MOG_35–55_ powder was dissolved in PBS at a concentration of 5 mg/mL, and PBS containing MOG_35–55_ was mixed 1:1 with complete Freund’s adjuvant. Tuberculin (H37Ra) was added to make a final concentration of 4 mg/mL. Mixing and emulsification were performed with a disperser, and the whole process was carried out on ice. A total of 0.1 mL of the emulsion was injected into 4 different sites on the posterior side of the back of the mice near the spine. Pertussis toxin (500 μg) was injected intraperitoneally into the mice at 0 and 48 hours of immunization. The status of the mice was observed daily, and the clinical scores were recorded as no disease, 0; tail paralysis, 1; paralysis of 1 side of the hind limb, 2; total paralysis of the hind limb, 3; complete paralysis of the limb, 4; and death, 5.

### Adoptive transfer.

The recipient mice were put into an x-ray irradiator and irradiated with 5 Gy, and the cells obtained from CD4^+^ T cell priming with MOG_35–55_ were injected into the tail vein of the recipient mice within 4–6 hours after irradiation. Each mouse was injected with 100 μL of approximately 1 × 10^7^ cells. The status of the mice was observed daily, and clinical scores were recorded.

### Isolation and analysis of CNS inflammatory cells.

After anesthetizing the mice, perfused with PBS, the mouse brain tissue was removed and placed in complete DMEM, cut with scissors, and processed into a single-cell suspension with a handheld homogenizer. The cell suspension was mixed with 100% Percoll (10× Percoll/PBS = 9:1) and mixed well until the concentration of Percoll reached 30%. The suspension was transferred to a 15 mL centrifuge tube, with 70% Percoll in the upper layer and the cell suspension in the lower layer. Density gradient centrifugation at 500*g* was carried out for 20 minutes; after centrifugation was completed, the third layer of cells was aspirated and transferred to a new 15-mL centrifugation tube. Then, 10 mL of complete medium was added, inverted, and spun at 500*g* for 5 minutes; the supernatant was discarded, and the inflammatory cells in the precipitate were used for brain tissue infiltration. The total cell number was determined by a cell counter, and then the proportion of different kinds of cells was detected by flow cytometry. The whole procedure was performed on ice.

### Determination of soluble and polymerized tubulin fractions.

Cells were lysed with MT buffer (80 mM PIPES, 1 mM MgCl_2_, 1 mM EGTA, protease inhibitor, and PMSF, pH 6.8) for 5 minutes at 37°C. Then, 0.5% Triton X-100 and 1 μM taxol were added, and centrifugation was carried out at 17,400*g* for 15 minutes at room temperature. The supernatant was denatured with sampling buffer as soluble microtubule protein fraction(s). The pellet was resuspended with cold MT buffer supplemented with 1% Triton X-100 and placed on ice for 15 minutes to depolymerize the microtubules. After centrifugation at 15,000*g* for 10 minutes, the supernatant was denatured and used as the polymerized microtubule fraction (P) in immunoblotting analysis.

### T cell activation.

For human TCR stimulation, T cells were cultured on plates coated with anti-CD3 and anti-CD28 (5 μg/mL) for 0–30 minutes. For T cell proliferation and IL-2 assay, T cells were stained with 0.5 μM CFSE for 5 minutes and then washed twice with PBS. T cells were cultured on plates coated with anti-CD3 and anti-CD28 (1 μg/mL) and cultured for 48 hours.

### PLAs.

To detect protein-protein interactions in situ, we performed PLAs using the Duolink In Situ Red Starter Kit (Sigma-Aldrich) following the manufacturer’s instructions. Briefly, cells were cultured on glass coverslips, fixed with 4% paraformaldehyde for 15 minutes at room temperature, and permeabilized with 0.2% Triton X-100 for 10 minutes. After washing, cells were blocked with Duolink blocking solution for 1 hour at 37°C in a humidified chamber.

Primary antibodies raised in different species were incubated overnight at 4°C. After washing, species-specific PLA probes (anti-rabbit PLUS and anti-mouse MINUS) were applied and incubated for 1 hour at 37°C. Ligation and amplification steps were performed using the provided ligase and polymerase, each for 30 and 100 minutes, respectively, at 37°C. Signal amplification was visualized as red fluorescent puncta, indicating close proximity (<40 nm) of the target proteins.

Slides were mounted with Duolink mounting medium containing DAPI for nuclear staining. Images were captured using a confocal microscope (Zeiss LSM 980). Negative controls without ANKRD55 primary antibodies were included to ensure signal specificity.

### Isolation of PBMCs from whole blood.

PBS was added to fresh anticoagulated whole blood according to a 1:1 ratio and mixed well. Ficoll was added to a sterile centrifuge tube, diluted with whole blood to the top of the Ficoll level (Ficoll/diluted whole blood = 1:2), keeping the interface between the 2 levels clear, and centrifuged at 800*g* for 20 minutes at room temperature. After centrifugation, the plasma layer was discarded, and the PBMC layer was carefully aspirated and transferred to a 15 mL centrifuge tube. The cells were resuspended by adding 10 mL of PBS to the tube and centrifuging at 300*g* for 10 minutes at room temperature, after which the supernatant was discarded. The procedure was repeated 2 times.

### Detection of immune synapses by flow cytometry and microscopy.

To assess immune synapse formation, Raji cells were labeled with CellTracker blue fluorescent probe (CMAC) for 5 minutes, followed by thorough washing to remove excess dye, and subsequently stimulated with SEE for 30 minutes. Jurkat cells were labeled with CMTPX for 30 minutes and similarly washed to remove residual dye. The 2 cell types were then coincubated for 20 minutes, and cell-cell conjugates were analyzed by flow cytometry.

For imaging-based analysis, Jurkat cells were stained with CMAC for 30 minutes and washed to eliminate excess dye. Raji cells were stimulated with SEE for 30 minutes, and unbound antigen was removed by washing. Jurkat and Raji cells were then cocultured for 30 minutes and transferred onto poly-d-lysine–coated slides for microscopy.

### Statistics.

All analyses were conducted using Prism 10.1.2 (GraphPad Software). Unpaired *t* tests were performed when comparing between 2 independent groups. For multiple comparisons between 2 groups, multiple unpaired *t* tests were performed with FDR correction using the 2-stage step-up method of Benjamini, Krieger, and Yekutieli. One-way ANOVA with appropriate multiple-comparison tests was performed when comparing more than 2 independent groups. Two-way ANOVA with repeated measures and appropriate multiple-comparison tests was performed when comparing 3 or more independent groups requiring clinical symptom evaluation with consistent observations. Values are presented as mean ± SEM. The significance threshold was set as *P* < 0.05.

### Study approval.

Human samples and animal experiments were conducted in accordance with the ethical standards of the Institutional Review Board and the Institutional Animal Care and Use Committee. Clinical sample collection and animal studies were approved by the Ethics Committee of Sichuan Provincial People’s Hospital.

### Data availability.

All data supporting the findings of this study are included in this article or in the Supporting Data Values file. No new code or algorithms were generated. The raw sequencing data supporting the findings of this study have been deposited in the National Center for Biotechnology Information Sequence Read Archive under BioProject accession number PRJNA1294800. The single-cell RNA-Seq data used in this study were obtained from the GEO database under accession number GSE138266. All of the data within the paper are available upon request from the authors.

## Author contributions

CJZ and MJ designed the study. C Wu, XY, YL, ZC, RC, GD, WW, MG, ZL, and ZL performed experiments. C Wu analyzed data. JF, ZC, YG, BH, and RC assisted with experiments. TP, C Wang, and CJZ wrote the original draft of the manuscript. LDCM and LMZ reviewed and edited the manuscript. All authors made a direct and intellectual contribution to this topic and approved the manuscript for publication.

## Supplementary Material

Supplemental data

Unedited blot and gel images

Supporting data values

## Version 1 10/15/2025

Electronic publication
