# Supplementary material for: ANKRD55 is a key regulator of T cell inflammation in multiple sclerosis
Source: J Clin Invest. 2025 Oct 15;135(20):e195214. doi: 10.1172/JCI195214 (PMC12520676; doi:10.1172/JCI195214)
Supplement: Supplemental data [file jci-135-195214-s205.pdf]

Figure S1

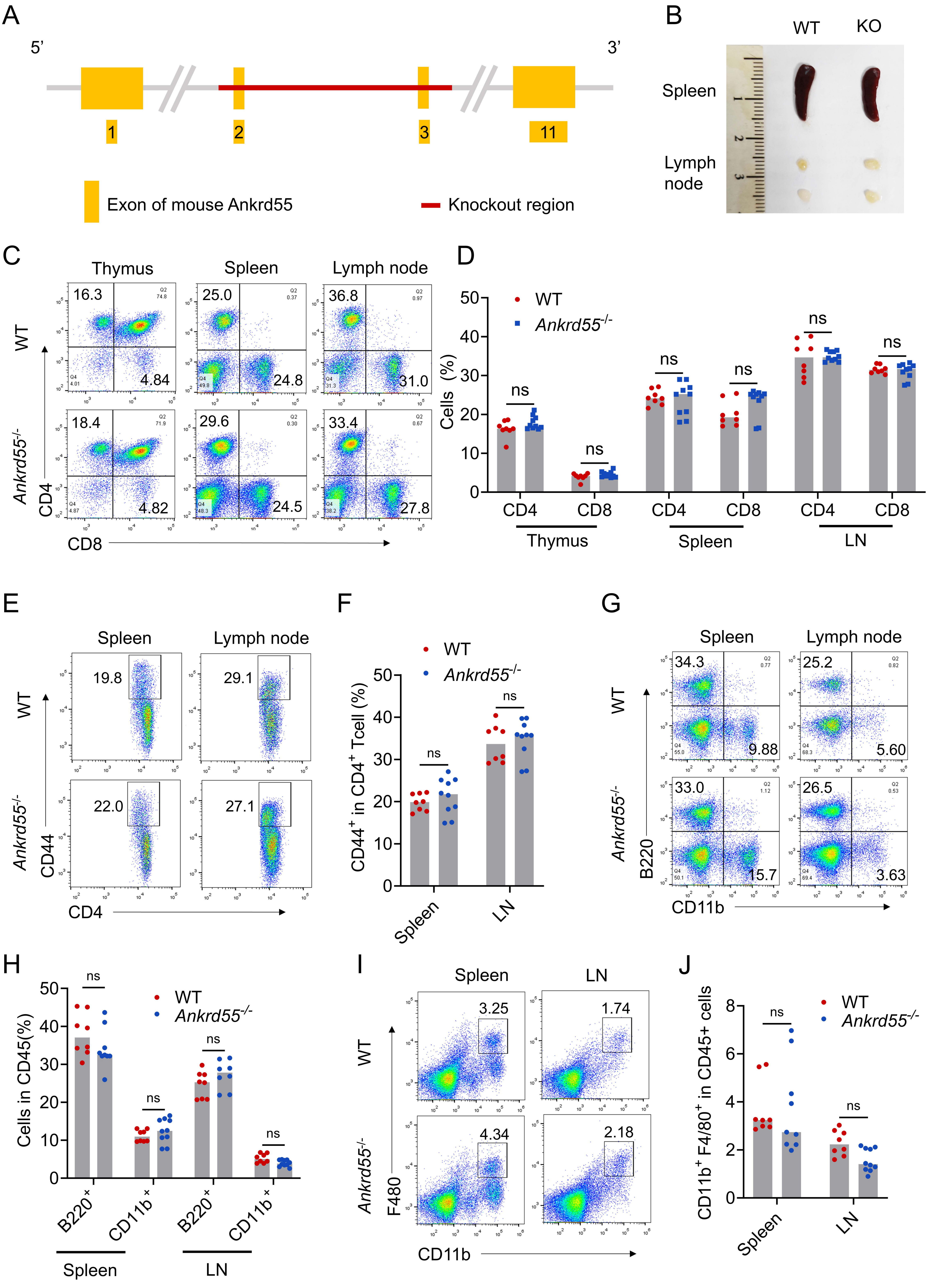
A
5'
3'
1
2
3
11
Exon of mouse Ankrd55
Knockout region
B
WT
KO
Spleen
Lymph node
C
Thymus
Spleen
Lymph node
WT
Ankrd55-/-
CD4
CD8
16.3
4.84
25.0
24.8
36.8
31.0
18.4
4.82
29.6
24.5
33.4
27.8
D
WT
Ankrd55-/-
Cells (%)
ns
CD4
CD8
Thymus
Spleen
LN
E
Spleen
Lymph node
WT
Ankrd55-/-
CD44
CD4
19.8
29.1
22.0
27.1
F
WT
Ankrd55-/-
CD44+ in CD4+ Tcell (%)
ns
Spleen
LN
G
Spleen
Lymph node
WT
Ankrd55-/-
B220
CD11b
34.3
9.88
25.2
5.60
33.0
15.7
26.5
3.63
H
Cells in CD45(%)
WT
Ankrd55-/-
ns
B220+
CD11b+
Spleen
LN
I
Spleen
LN
WT
Ankrd55-/-
F480
CD11b
3.25
1.74
4.34
2.18
J
CD11b+ F4/80+ in CD45+ cells
WT
Ankrd55-/-
ns
Spleen
LN

Figure S2

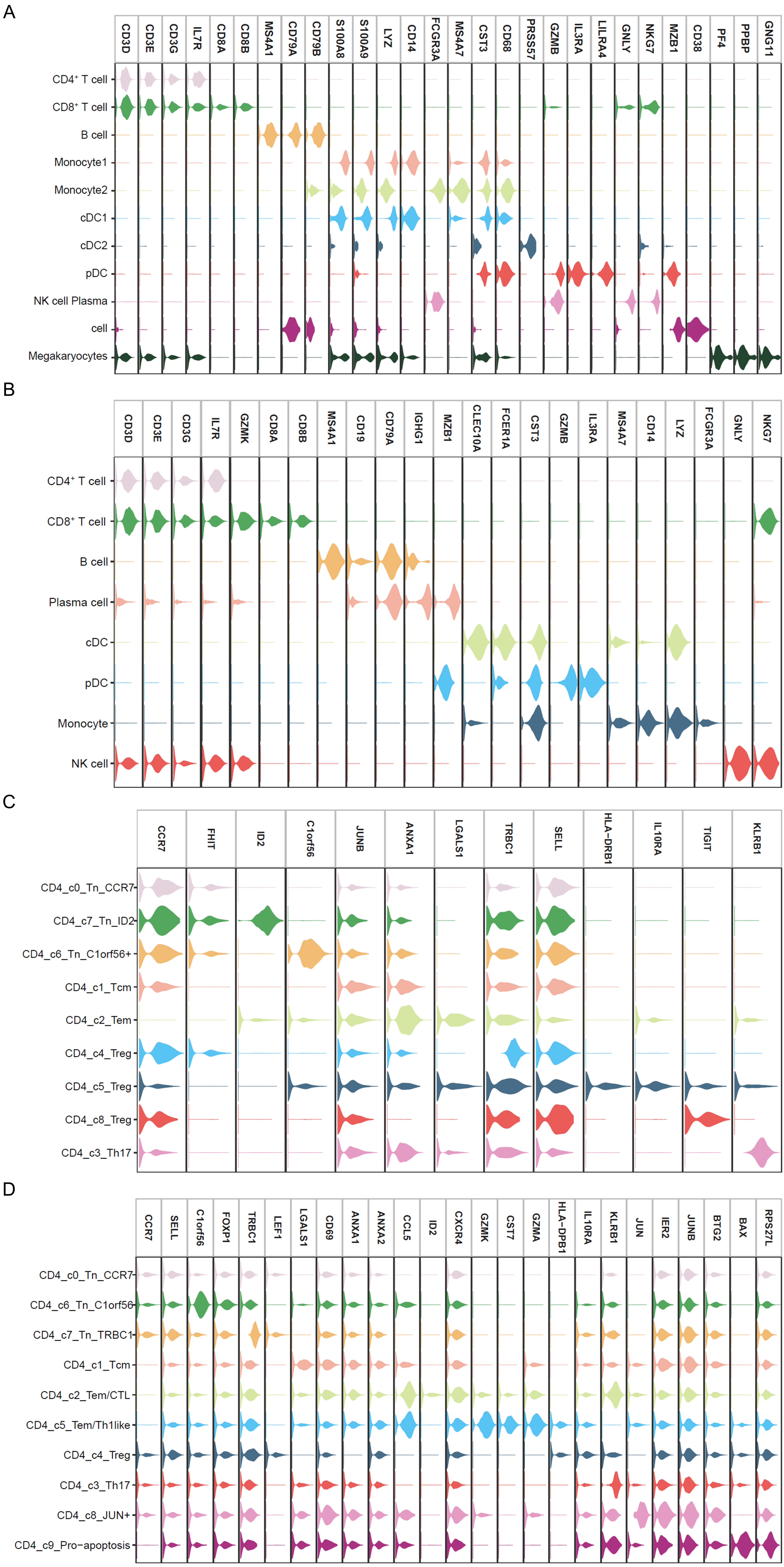

A
CD3D
CD3E
CD3G
IL7R
CD8A
CD8B
MS4A1
CD79A
CD79B
S100A8
S100A9
LYZ
CD14
FCGR3A
MS4A7
CST3
CD68
PRSS57
GZMB
IL3RA
LILRA4
GNLY
NKG7
MZB1
CD38
PF4
PPBP
GNG11
CD4+ T cell
CD8+ T cell
B cell
Monocyte1
Monocyte2
cDC1
cDC2
pDC
NK cell Plasma
cell
Megakaryocytes
B
CD3D
CD3E
CD3G
IL7R
GZMK
CD8A
CD8B
MS4A1
CD19
CD79A
IGHG1
MZB1
CLEC10A
FCER1A
CST3
GZMB
IL3RA
MS4A7
CD14
LYZ
FCGR3A
GNLY
NKG7
CD4+ T cell
CD8+ T cell
B cell
Plasma cell
cDC
pDC
Monocyte
NK cell
C
CCR7
FHIT
ID2
C1orf56
JUNB
ANXA1
LGALS1
TRBC1
SELL
HLA−DRB1
IL10RA
TIGIT
KLRB1
CD4_c0_Tn_CCR7
CD4_c7_Tn_ID2
CD4_c6_Tn_C1orf56+
CD4_c1_Tcm
CD4_c2_Tem
CD4_c4_Treg
CD4_c5_Treg
CD4_c8_Treg
CD4_c3_Th17
D
CCR7
SELL
C1orf56
FOXP1
TRBC1
LEF1
LGALS1
CD69
ANXA1
ANXA2
CCL5
ID2
CXCR4
GZMK
CST7
GZMA
HLA−DPB1
IL10RA
KLRB1
JUN
IER2
JUNB
BTG2
BAX
RPS27L
CD4_c0_Tn_CCR7
CD4_c6_Tn_C1orf56
CD4_c7_Tn_TRBC1
CD4_c1_Tcm
CD4_c2_Tem/CTL
CD4_c5_Tem/Th1like
CD4_c4_Treg
CD4_c3_Th17
CD4_c8_JUN+
CD4_c9_Pro−apoptosis

Figure S3

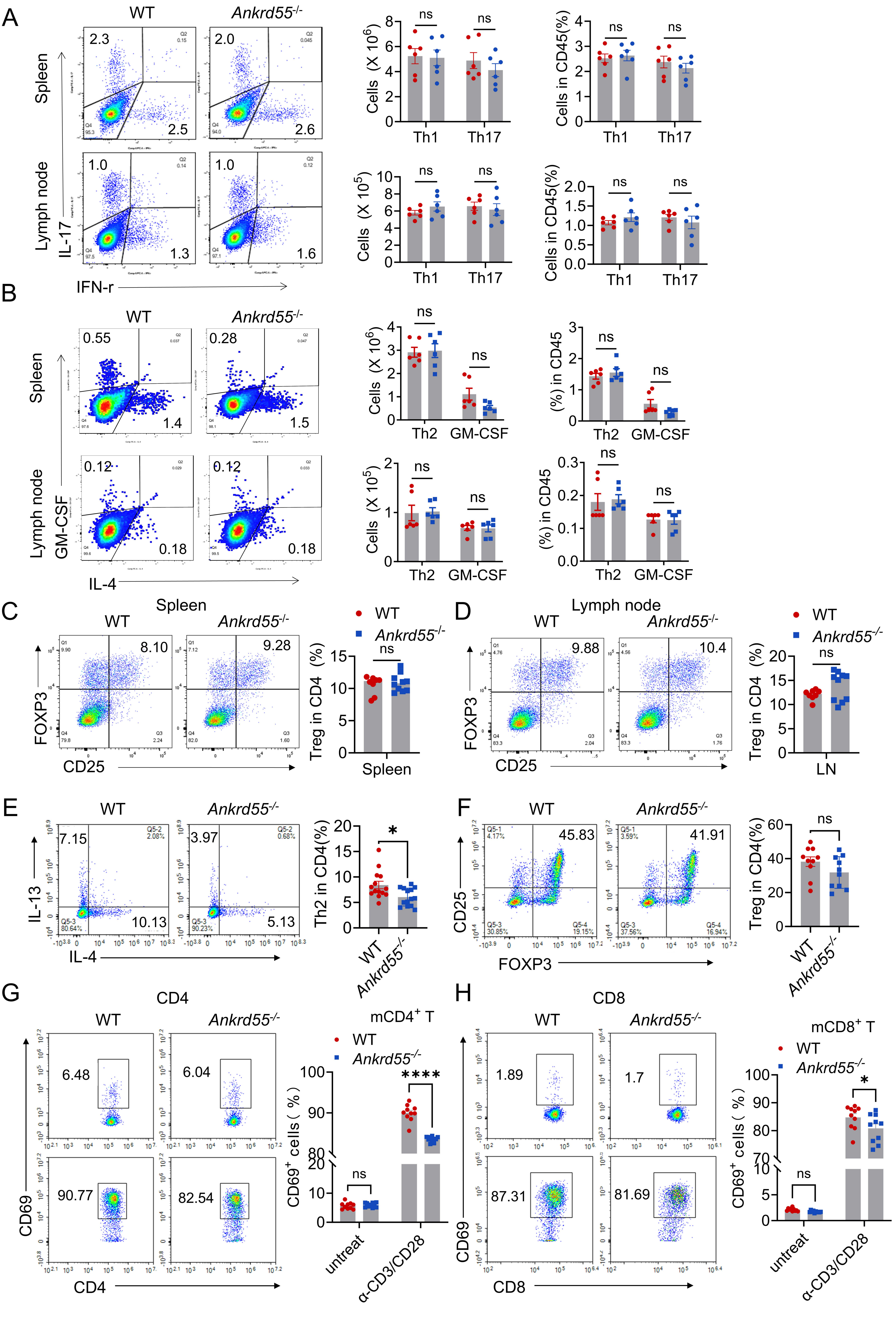
A
WT
Ankrd55-/-
Spleen
Lymph node
IL-17
IFN-r
2.3
2.5
2.0
2.6
1.0
1.3
1.0
1.6
Cells (X 10^6)
Cells in CD45(%)
Cells (X 10^5)
Th1
Th17
ns
B
GM-CSF
IL-4
0.55
1.4
0.28
1.5
0.12
0.18
Th2
(%) in CD45
C
Spleen
FOXP3
CD25
8.10
9.28
Treg in CD4 (%)
D
Lymph node
9.88
10.4
LN
E
IL-13
7.15
10.13
3.97
5.13
Th2 in CD4(%)
*
F
45.83
41.91
Treg in CD4(%)
G
CD4
CD69
6.48
6.04
90.77
82.54
mCD4+ T
CD69+ cells (%)
****
untreat
α-CD3/CD28
H
CD8
1.89
1.7
87.31
81.69
mCD8+ T

Figure S4

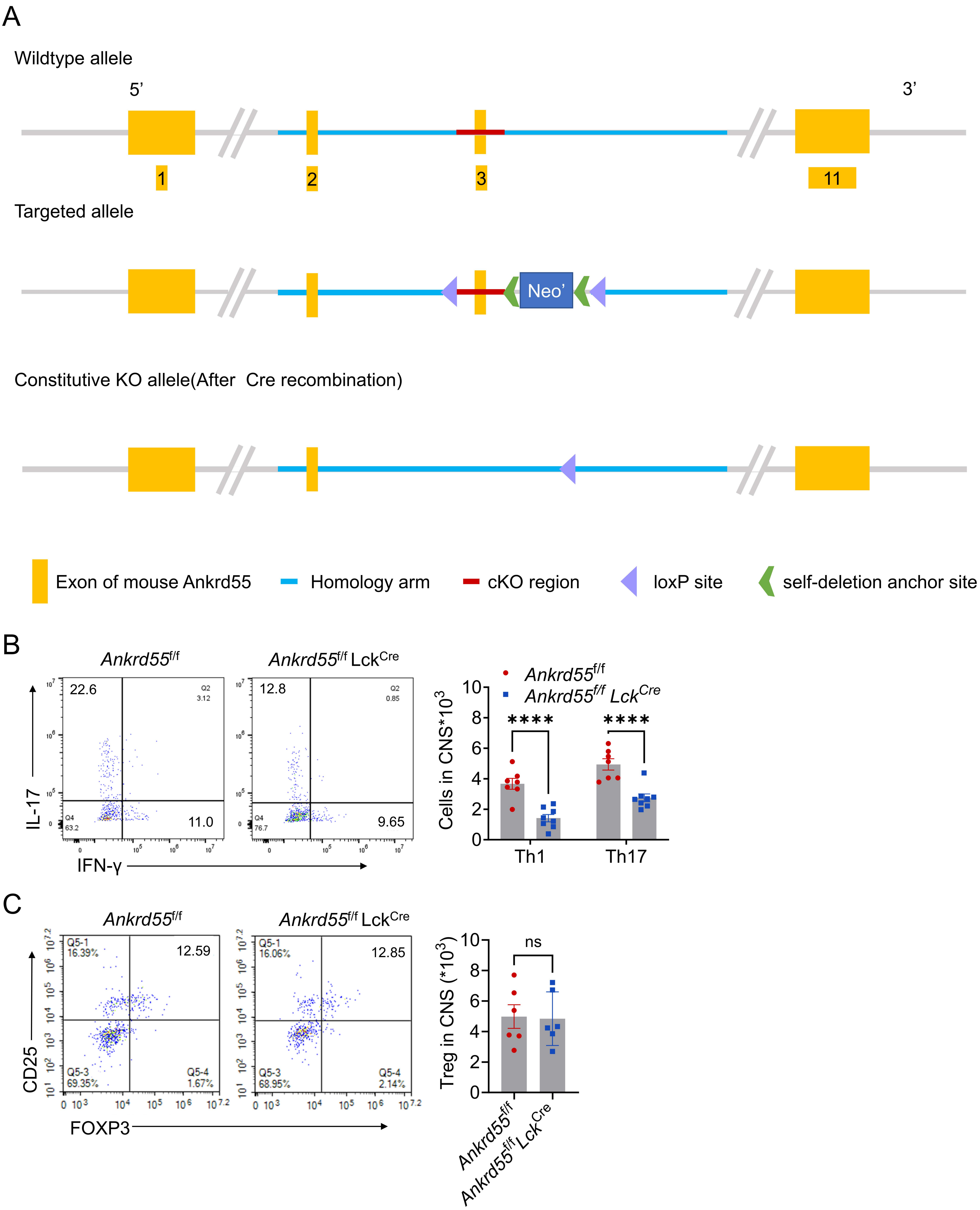

A
Wildtype allele
5’
3’
1
2
3
11
Targeted allele
Neo’
Constitutive KO allele(After Cre recombination)
Exon of mouse Ankrd55
Homology arm
cKO region
loxP site
self-deletion anchor site
B
Ankrd55f/f
Ankrd55f/f LckCre
22.6
Q2
3.12
Q4
63.2
11.0
12.8
Q2
0.85
Q4
76.7
9.65
IL-17
IFN-γ
Cells in CNS*10^3
****
****
Th1
Th17
C
Ankrd55f/f
Ankrd55f/f LckCre
Q5-1
16.39%
12.59
Q5-3
69.35%
Q5-4
1.67%
Q5-1
16.06%
12.85
Q5-3
68.95%
Q5-4
2.14%
CD25
FOXP3
Treg in CNS (*10^3)
ns
Ankrd55f/f
Ankrd55f/f LckCre

Figure S5

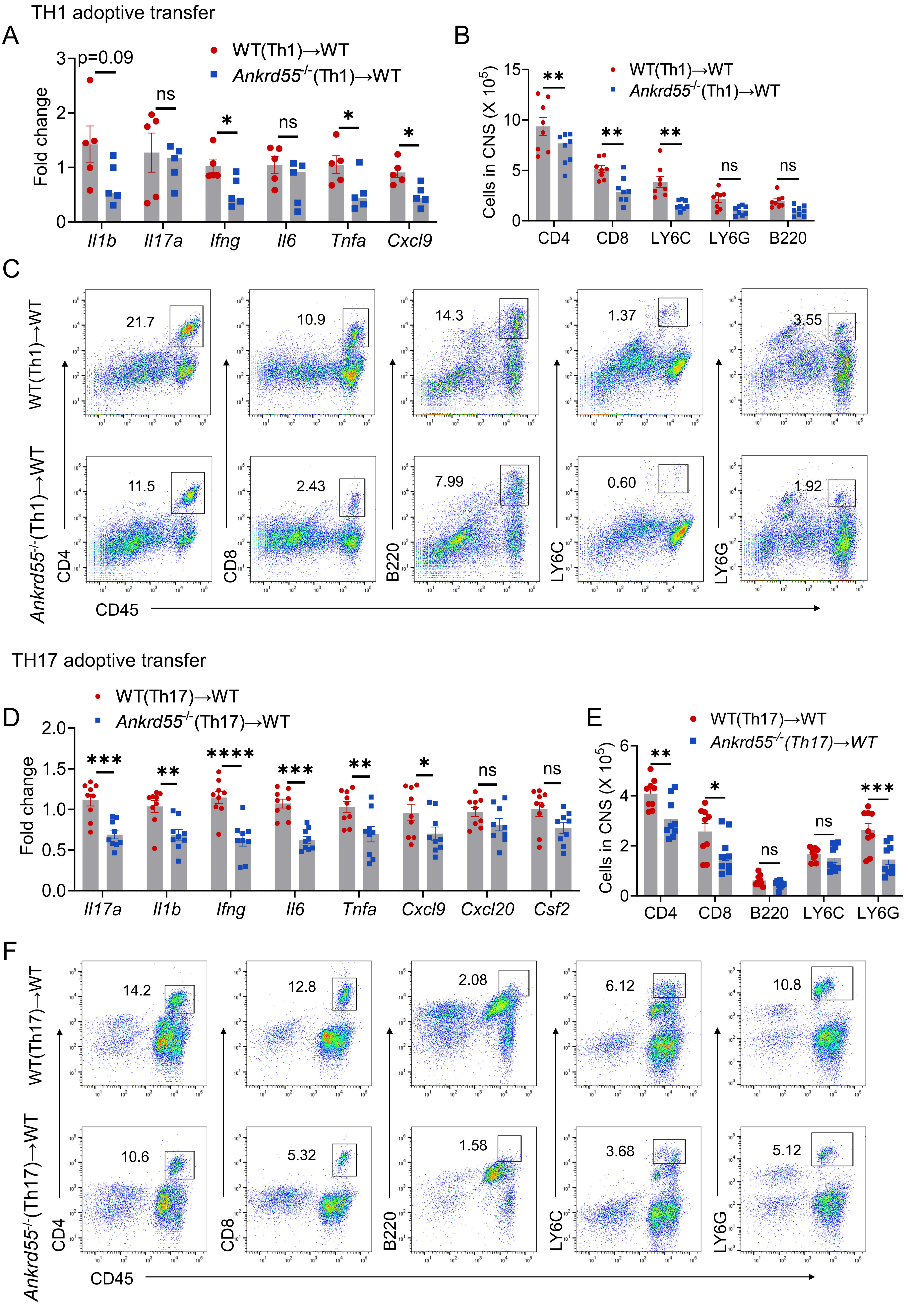
TH1 adoptive transfer
A
p=0.09
ns
*
ns
*
*
WT(Th1)→WT
Ankrd55-/-(Th1)→WT
Fold change
Il1b
Il17a
Ifng
Il6
Tnfa
Cxcl9
B
Cells in CNS (X 10^5)
**
**
**
ns
ns
CD4
CD8
LY6C
LY6G
B220
C
WT(Th1)→WT
Ankrd55-/-(Th1)→WT
21.7
10.9
14.3
1.37
3.55
11.5
2.43
7.99
0.60
1.92
CD4
CD8
B220
LY6C
LY6G
CD45
TH17 adoptive transfer
D
WT(Th17)→WT
Ankrd55-/-(Th17)→WT
Fold change
***
**
****
***
**
*
ns
ns
Il17a
Il1b
Ifng
Il6
Tnfa
Cxcl9
Cxcl20
Csf2
E
WT(Th17)→WT
Ankrd55-/-(Th17)→WT
Cells in CNS (X 10^5)
**
*
ns
ns
***
CD4
CD8
B220
LY6C
LY6G
F
WT(Th17)→WT
Ankrd55-/-(Th17)→WT
14.2
12.8
2.08
6.12
10.8
10.6
5.32
1.58
3.68
5.12
CD4
CD8
B220
LY6C
LY6G
CD45

Figure S6

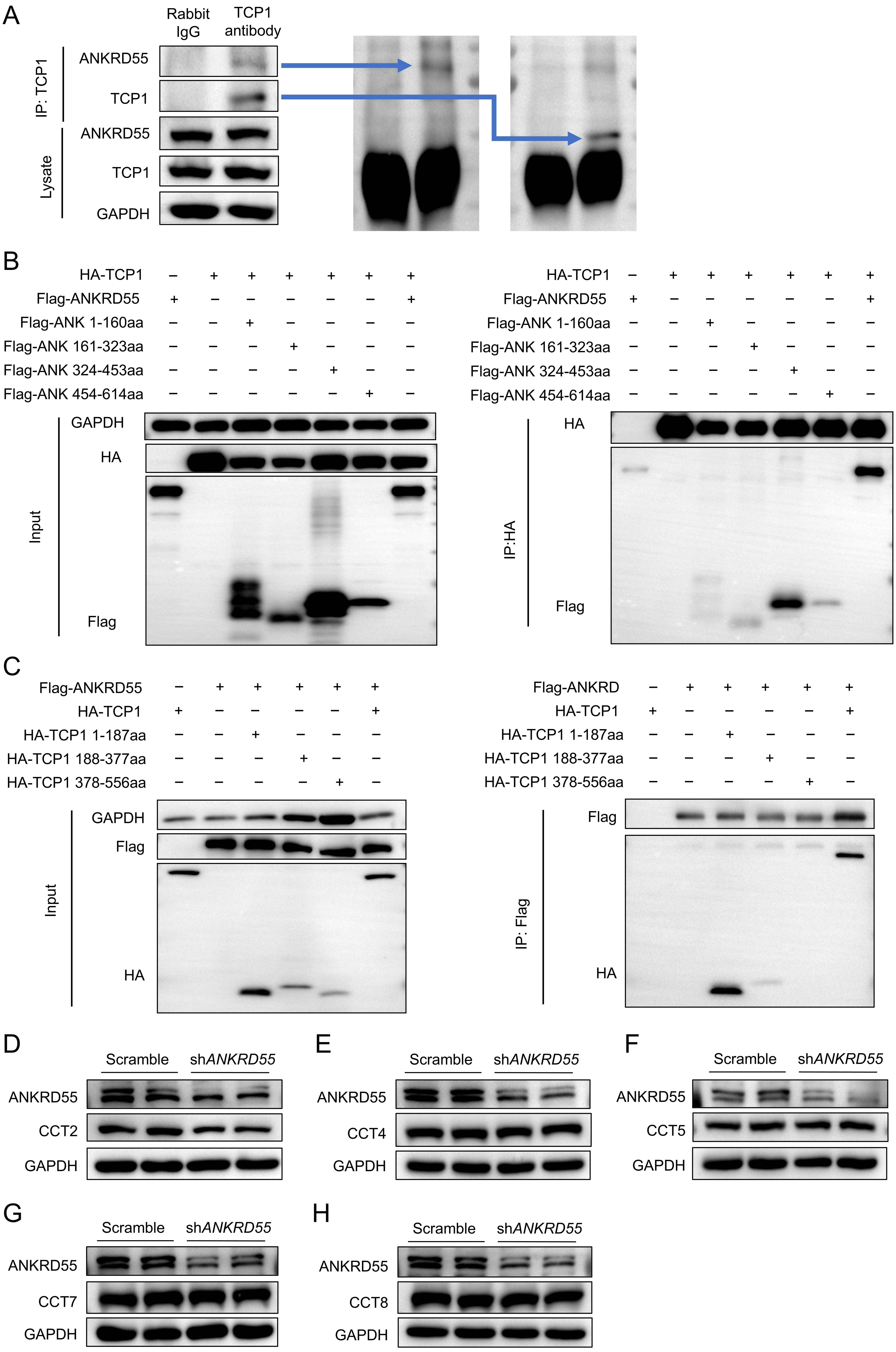

A
Rabbit IgG
TCP1 antibody
IP: TCP1
ANKRD55
TCP1
Lysate
ANKRD55
TCP1
GAPDH
B
HA-TCP1
Flag-ANKRD55
Flag-ANK 1-160aa
Flag-ANK 161-323aa
Flag-ANK 324-453aa
Flag-ANK 454-614aa
Input
GAPDH
HA
Flag
IP:HA
HA
Flag
C
Flag-ANKRD55
HA-TCP1
HA-TCP1 1-187aa
HA-TCP1 188-377aa
HA-TCP1 378-556aa
Input
GAPDH
Flag
HA
Flag-ANKRD
IP: Flag
Flag
HA
D
Scramble
shANKRD55
ANKRD55
CCT2
GAPDH
E
Scramble
shANKRD55
ANKRD55
CCT4
GAPDH
F
Scramble
shANKRD55
ANKRD55
CCT5
GAPDH
G
Scramble
shANKRD55
ANKRD55
CCT7
GAPDH
H
Scramble
shANKRD55
ANKRD55
CCT8
GAPDH

Figure S7

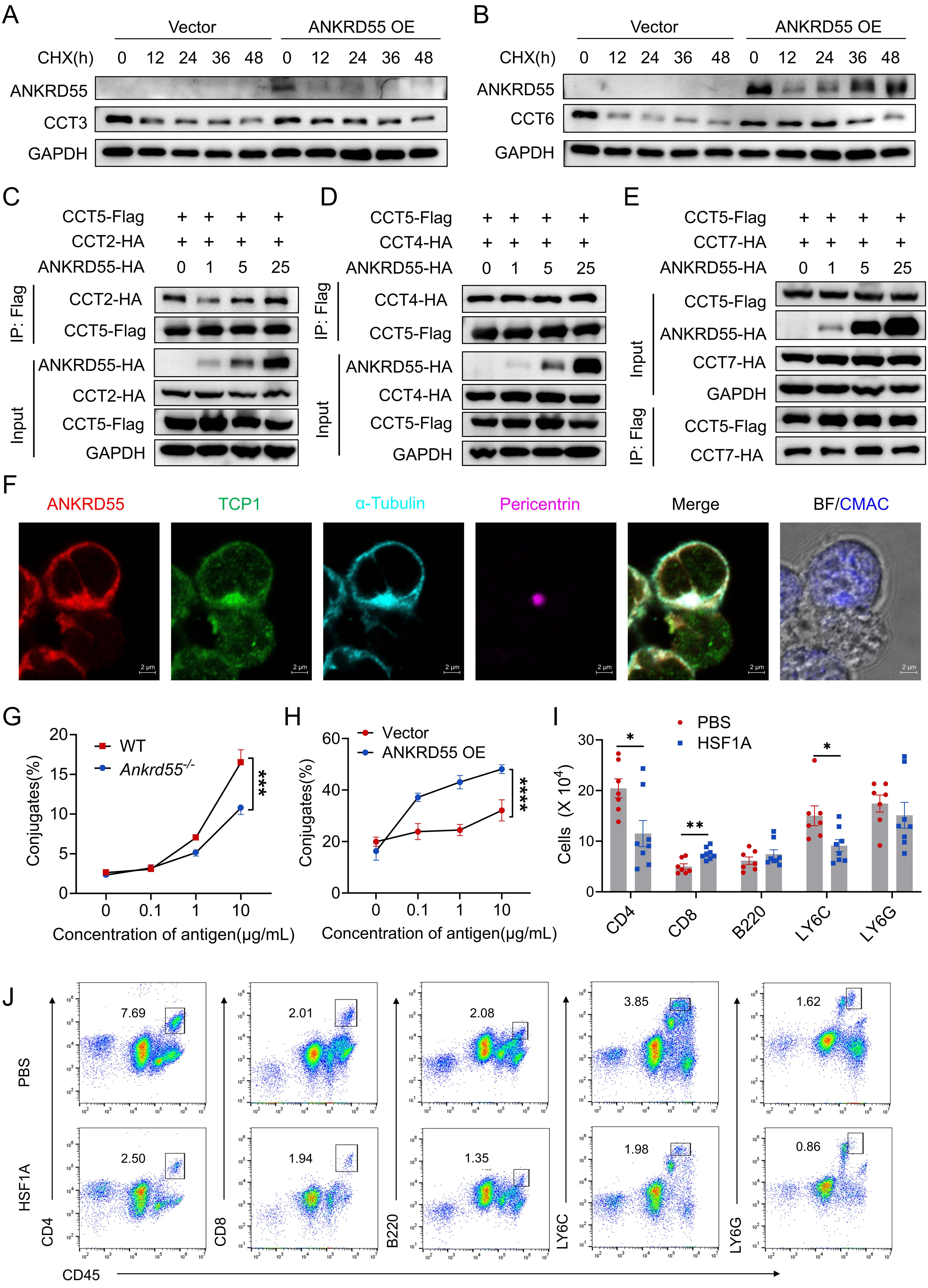

A
Vector
ANKRD55 OE
CHX(h) 0 12 24 36 48 0 12 24 36 48
ANKRD55
CCT3
GAPDH
B
Vector
ANKRD55 OE
CHX(h) 0 12 24 36 48 0 12 24 36 48
ANKRD55
CCT6
GAPDH
C
CCT5-Flag + + + +
CCT2-HA + + + +
ANKRD55-HA 0 1 5 25
IP: Flag
CCT2-HA
CCT5-Flag
Input
ANKRD55-HA
CCT2-HA
CCT5-Flag
GAPDH
D
CCT5-Flag + + + +
CCT4-HA + + + +
ANKRD55-HA 0 1 5 25
IP: Flag
CCT4-HA
CCT5-Flag
Input
ANKRD55-HA
CCT4-HA
CCT5-Flag
GAPDH
E
CCT5-Flag + + + +
CCT7-HA + + + +
ANKRD55-HA 0 1 5 25
Input
CCT5-Flag
ANKRD55-HA
CCT7-HA
GAPDH
IP: Flag
CCT5-Flag
CCT7-HA
F
ANKRD55
TCP1
α-Tubulin
Pericentrin
Merge
BF/CMAC
2 μm
G
WT
Ankrd55-/-
***
Conjugates(%)
Concentration of antigen(μg/mL)
H
Vector
ANKRD55 OE
****
Conjugates(%)
Concentration of antigen(μg/mL)
I
PBS
HSF1A
Cells (X 10^4)
CD4
CD8
B220
LY6C
LY6G
*
**
*
J
PBS
HSF1A
7.69
2.01
2.08
3.85
1.62
2.50
1.94
1.35
1.98
0.86
CD4
CD8
B220
LY6C
LY6G
CD45

Figure S8

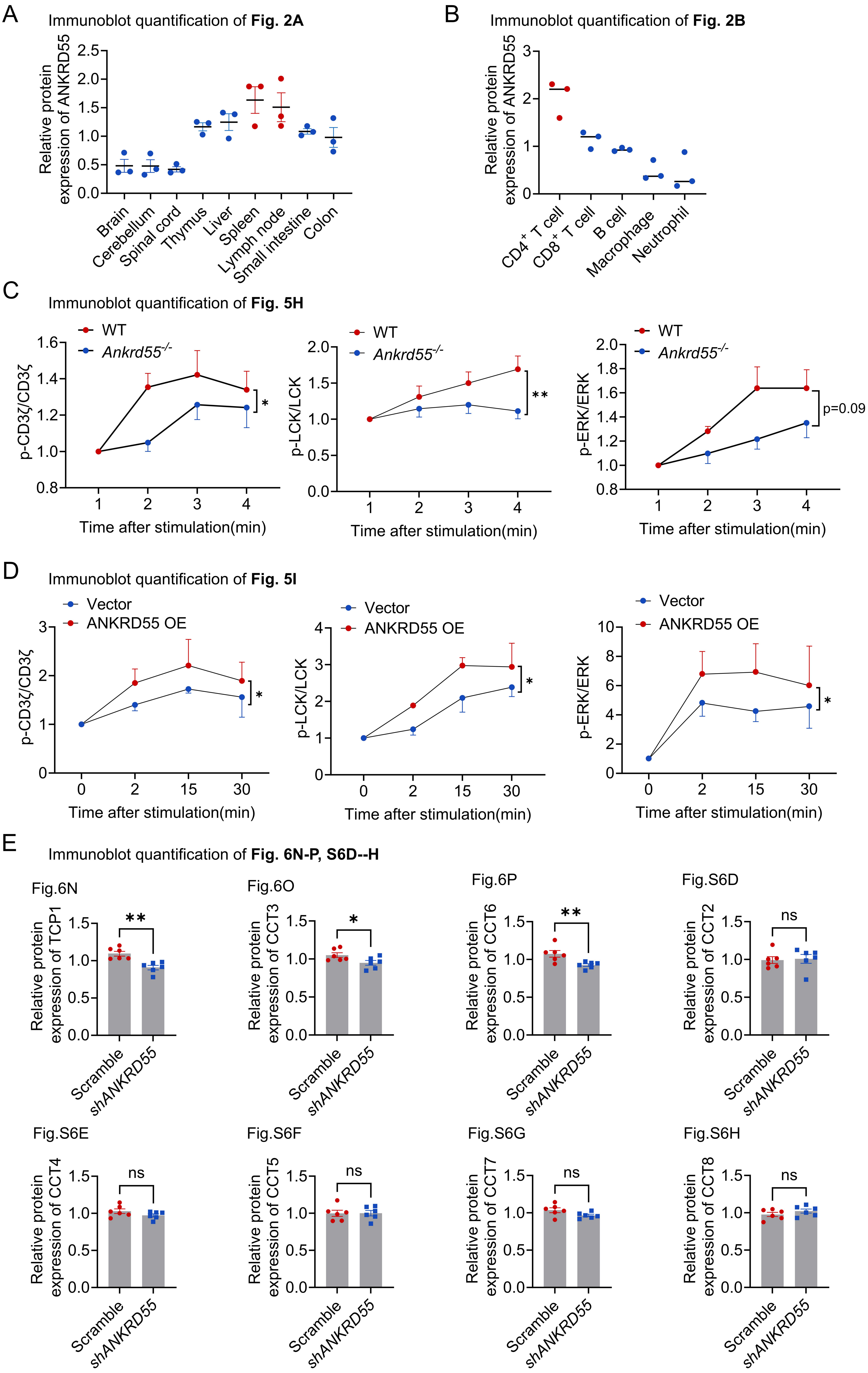
A
Immunoblot quantification of **Fig. 2A**
Relative protein expression of ANKRD55
2.5
2.0
1.5
1.0
0.5
0.0
Brain
Cerebellum
Spinal cord
Thymus
Liver
Spleen
Lymph node
Small intestine
Colon
B
Immunoblot quantification of **Fig. 2B**
Relative protein expression of ANKRD55
3
2
1
0
CD4+ T cell
CD8+ T cell
B cell
Macrophage
Neutrophil
C
Immunoblot quantification of **Fig. 5H**
WT
Ankrd55-/-
p-CD3ζ/CD3ζ
1.6
1.4
1.2
1.0
0.8
1
2
3
4
*
Time after stimulation(min)
WT
Ankrd55-/-
p-LCK/LCK
2.0
1.5
1.0
0.5
0.0
1
2
3
4
**
Time after stimulation(min)
WT
Ankrd55-/-
p-ERK/ERK
2.0
1.8
1.6
1.4
1.2
1.0
0.8
1
2
3
4
p=0.09
Time after stimulation(min)
D
Immunoblot quantification of **Fig. 5I**
Vector
ANKRD55 OE
p-CD3ζ/CD3ζ
3
2
1
0
0
2
15
30
*
Time after stimulation(min)
Vector
ANKRD55 OE
p-LCK/LCK
4
3
2
1
0
0
2
15
30
*
Time after stimulation(min)
Vector
ANKRD55 OE
p-ERK/ERK
10
8
6
4
2
0
0
2
15
30
*
Time after stimulation(min)
E
Immunoblot quantification of **Fig. 6N-P, S6D--H**
Fig.6N
Relative protein expression of TCP1
**
Fig.6O
Relative protein expression of CCT3
*
Fig.6P
Relative protein expression of CCT6
**
Fig.S6D
Relative protein expression of CCT2
ns
Fig.S6E
Relative protein expression of CCT4
ns
Fig.S6F
Relative protein expression of CCT5
ns
Fig.S6G
Relative protein expression of CCT7
ns
Fig.S6H
Relative protein expression of CCT8
ns
1.5
1.0
0.5
0.0
Scramble
shANKRD55

Figure S9

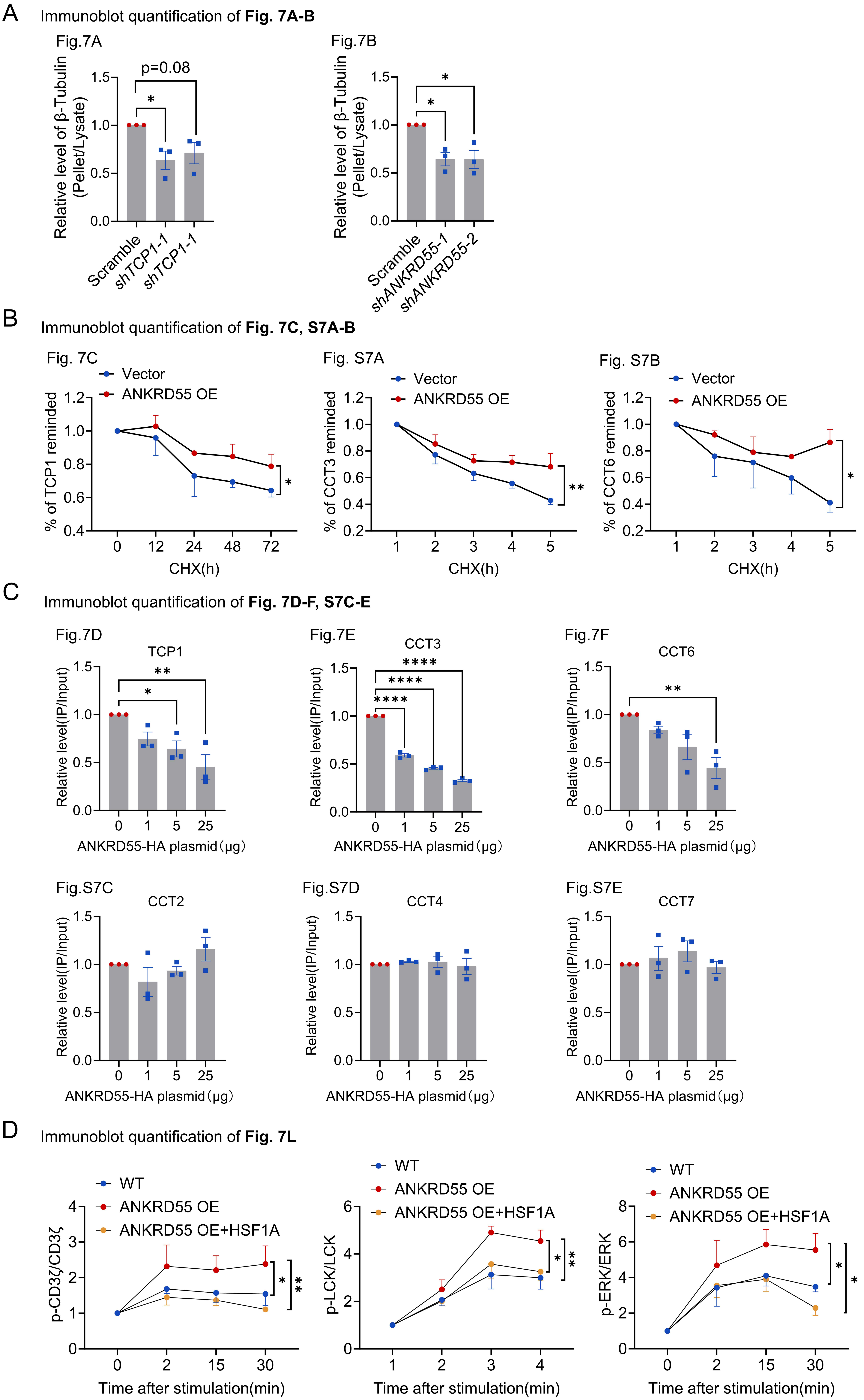
A
Immunoblot quantification of Fig. 7A-B
Fig.7A
Relative level of β-Tubulin (Pellet/Lysate)
p=0.08
*
Scramble
shTCP1-1
shTCP1-1
Fig.7B
*
*
Scramble
shANKRD55-1
shANKRD55-2
B
Immunoblot quantification of Fig. 7C, S7A-B
Fig. 7C
Vector
ANKRD55 OE
% of TCP1 reminded
CHX(h)
Fig. S7A
% of CCT3 reminded
**
Fig. S7B
% of CCT6 reminded
C
Immunoblot quantification of Fig. 7D-F, S7C-E
Fig.7D
TCP1
Fig.7E
CCT3
****
Fig.7F
CCT6
Relative level(IP/Input)
ANKRD55-HA plasmid (μg)
Fig.S7C
CCT2
Fig.S7D
CCT4
Fig.S7E
CCT7
D
Immunoblot quantification of Fig. 7L
WT
ANKRD55 OE
ANKRD55 OE+HSF1A
p-CD3ζ/CD3ζ
p-LCK/LCK
p-ERK/ERK
Time after stimulation(min)
