# Supplementary material for: ANKRD55 is a key regulator of T cell inflammation in multiple sclerosis
Source: J Clin Invest. 2025 Oct 15;135(20):e195214. doi: 10.1172/JCI195214 (PMC12520676; doi:10.1172/JCI195214)

Figure 1A

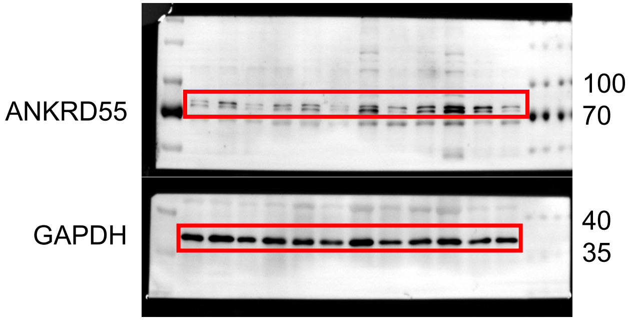

Figure 1B

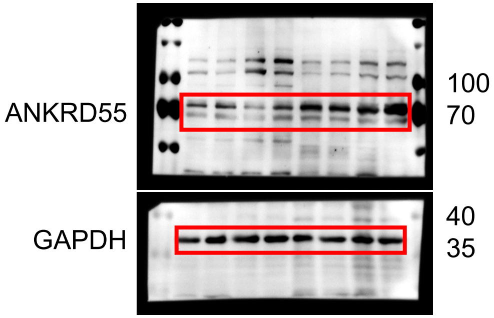

Figure 1C

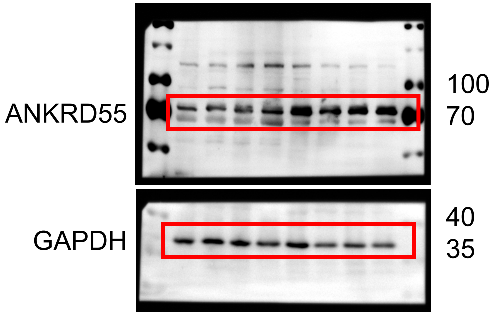

Figure 2A

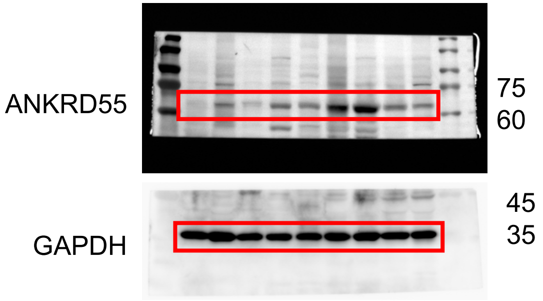

Figure 2B

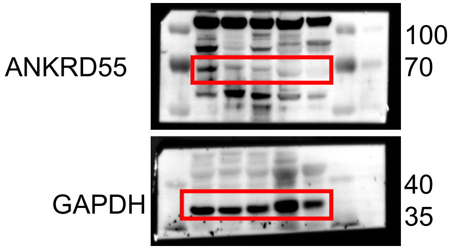

Figure 4A

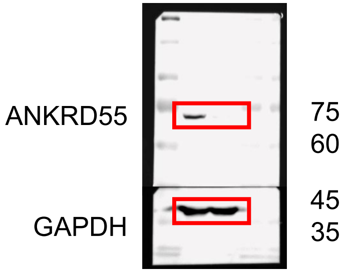

Figure 5H

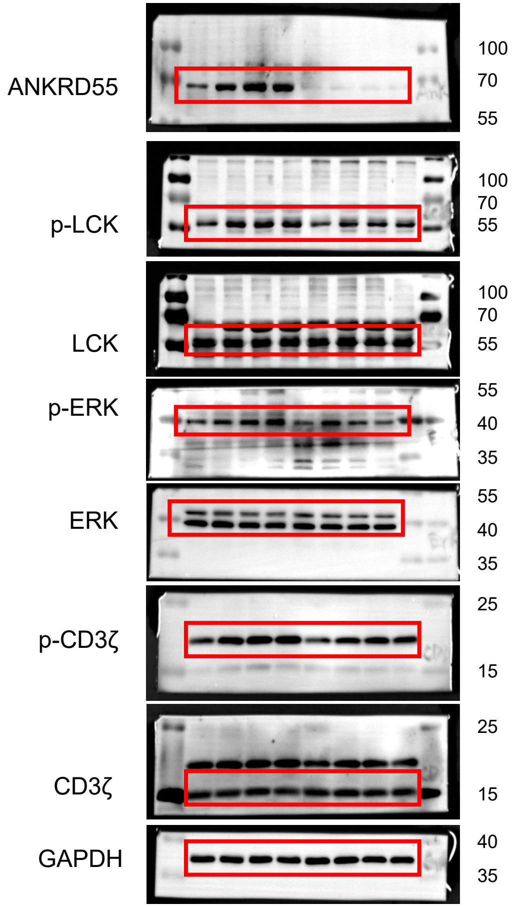

Figure 5I

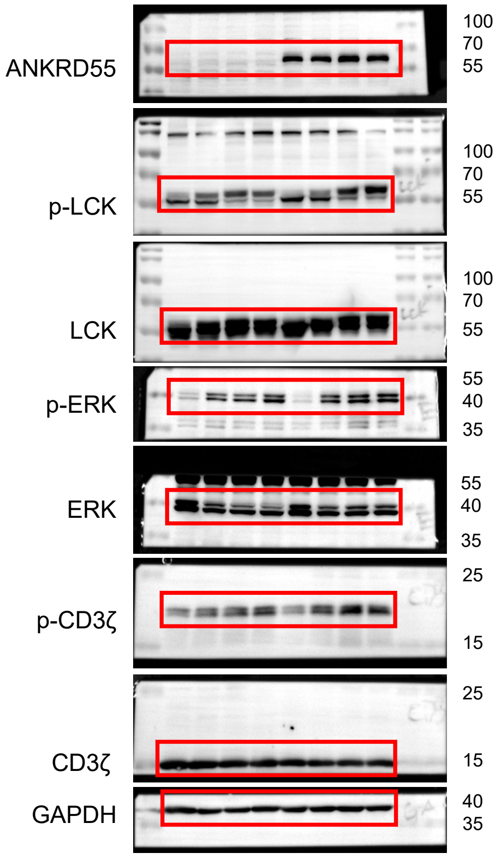

Figure 6A

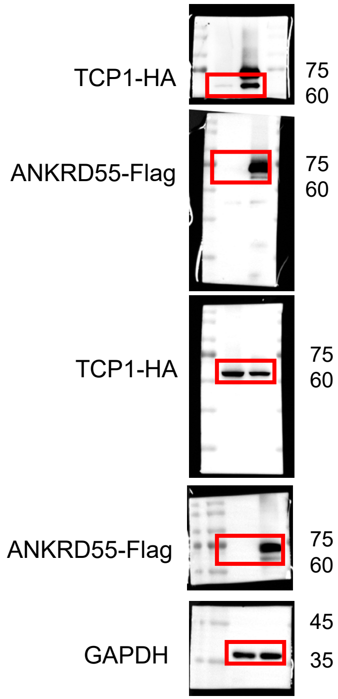

Figure 6B

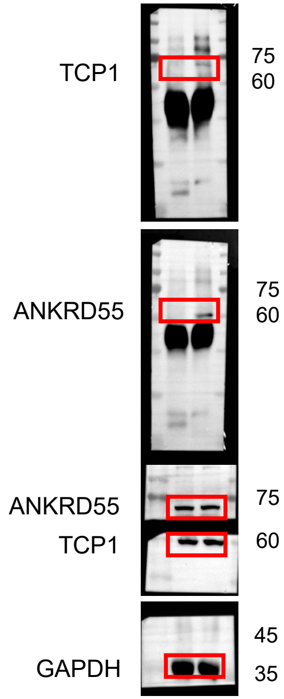

Figure 6E,F,G

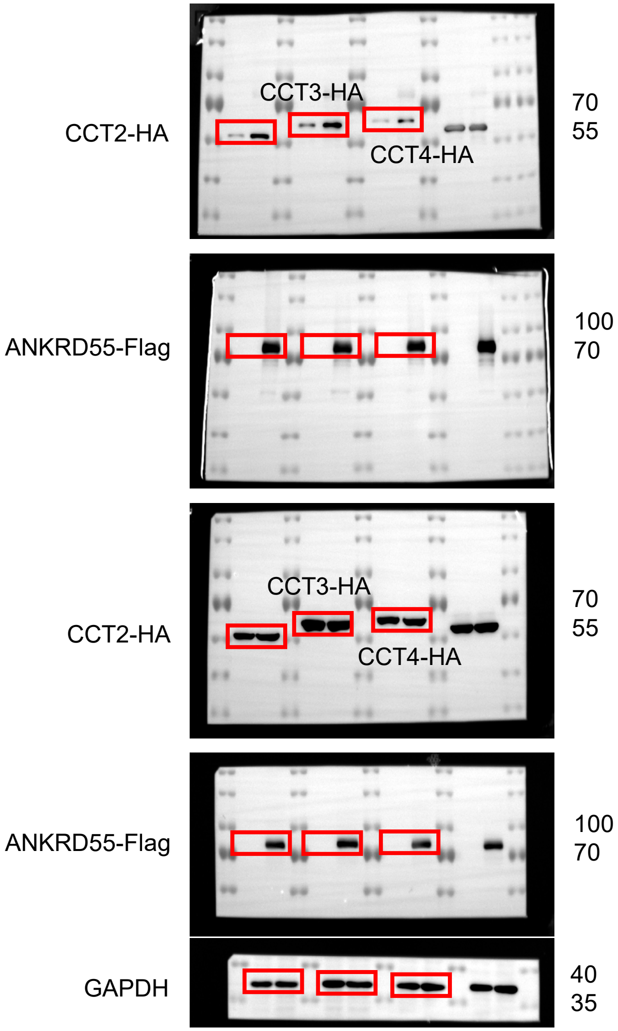

Figure 6H

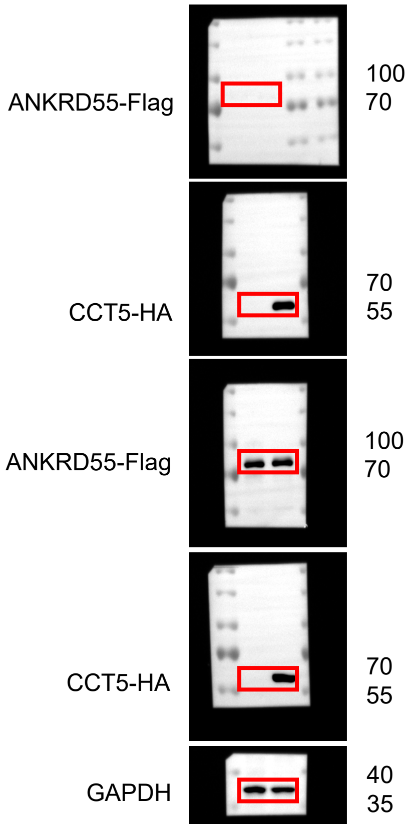

Figure 6I

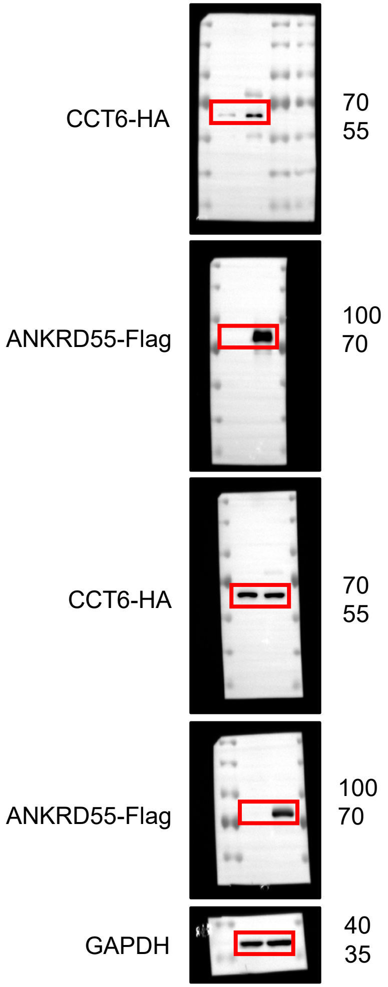

Figure 6J,K

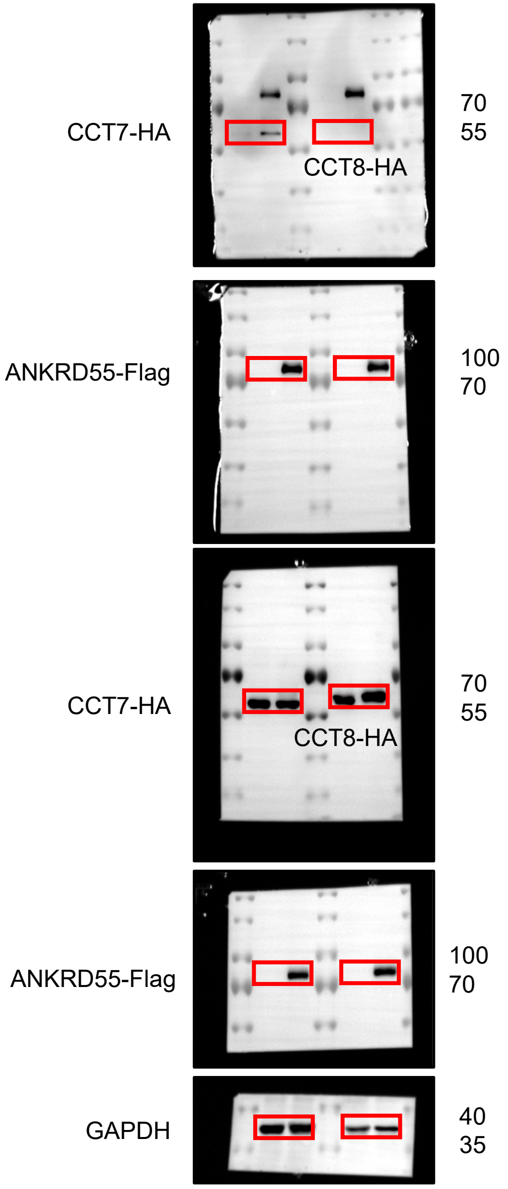

Figure 7A

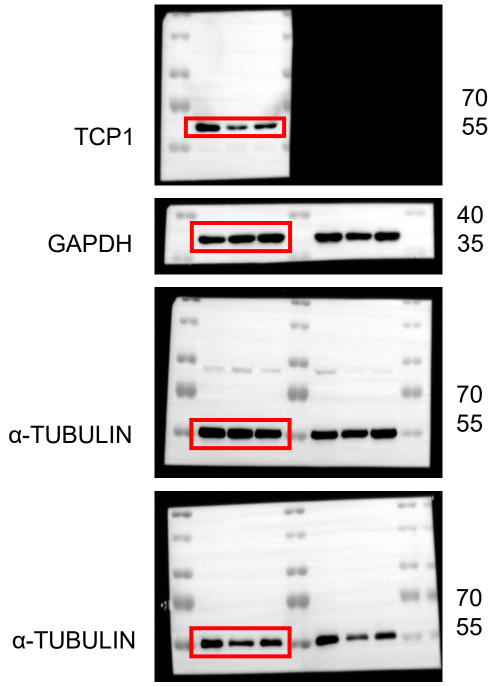

Figure 7B

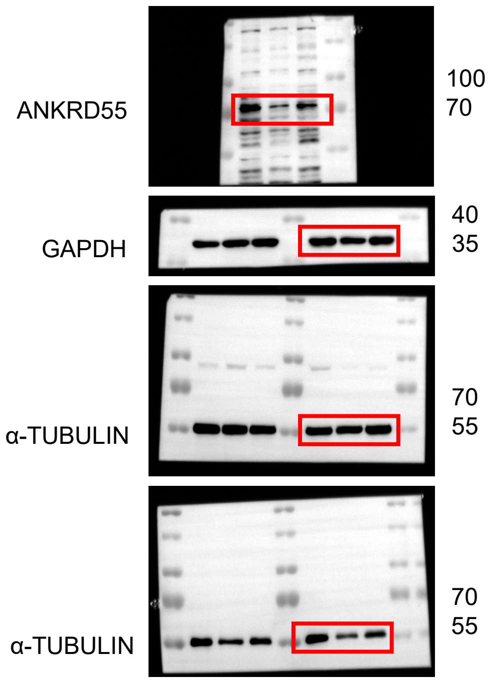

Figure 7C

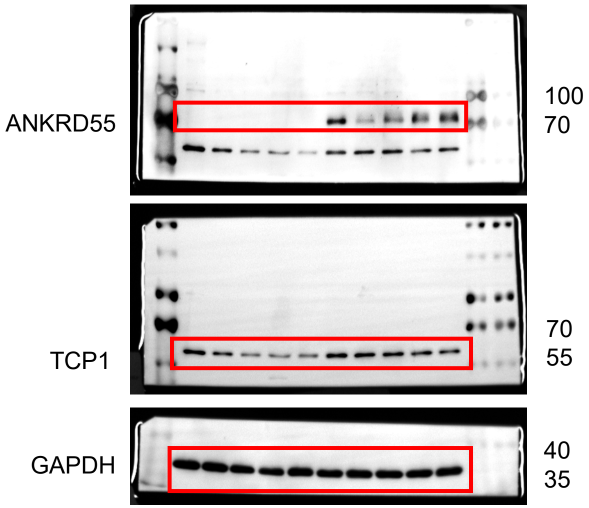

Figure 7D

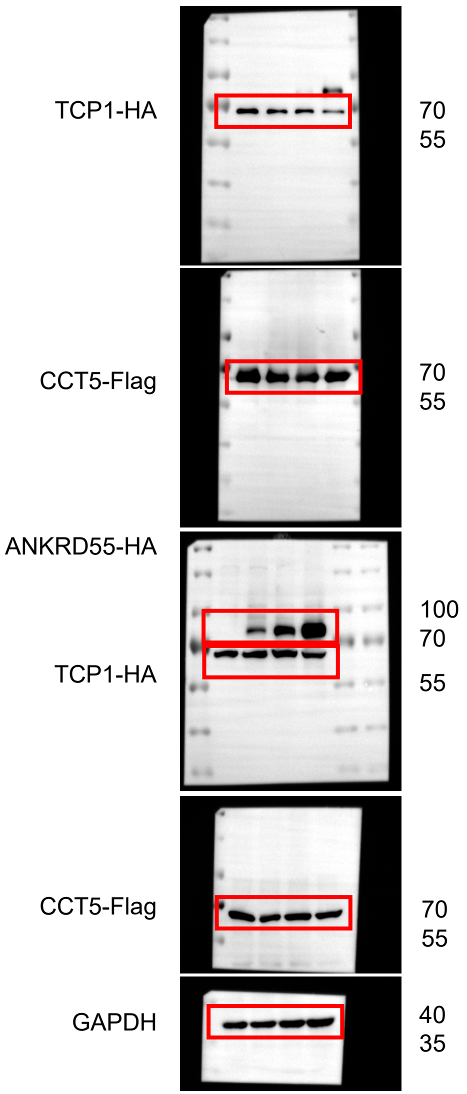

Figure 7E

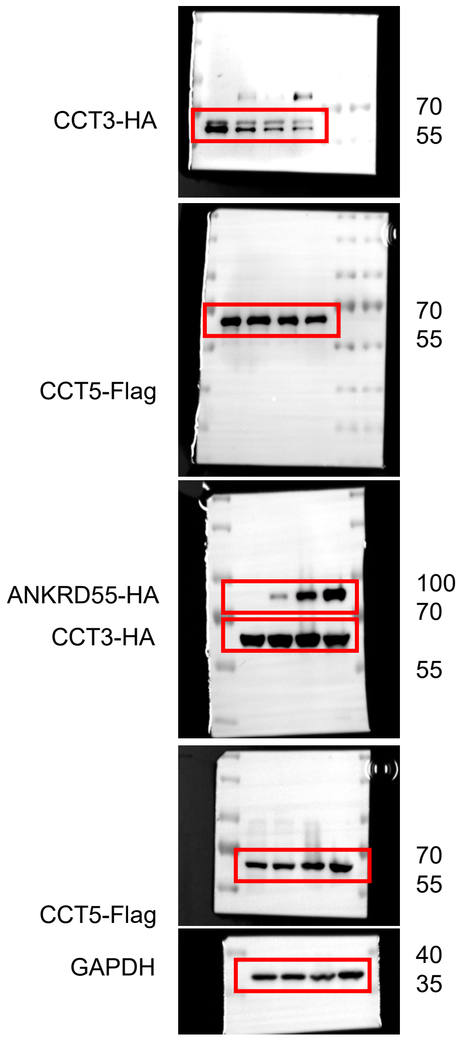

Figure 7F

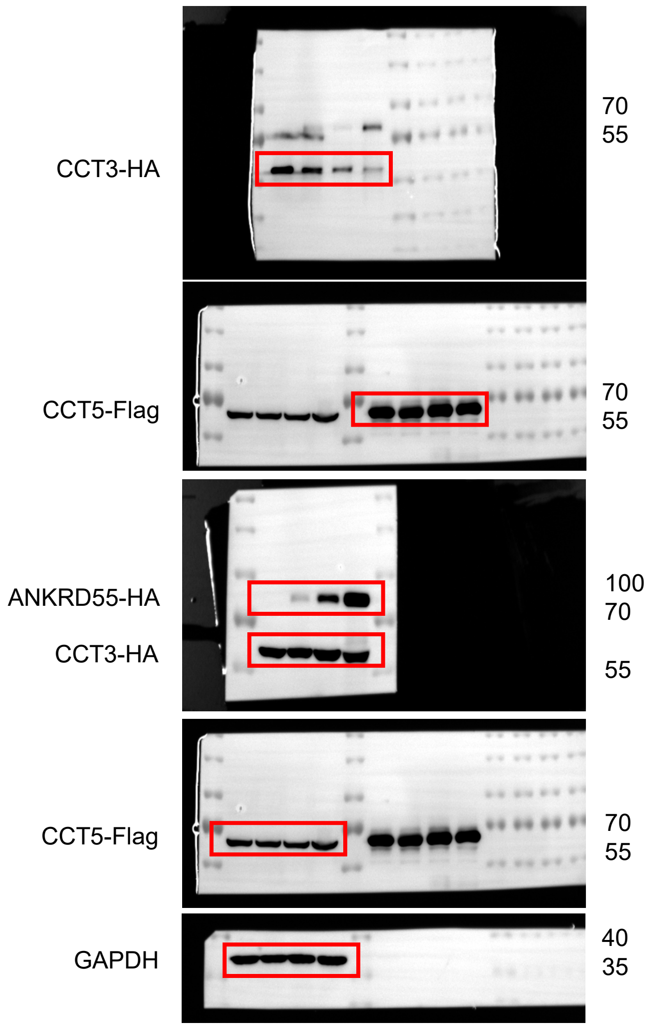

Figure 7K

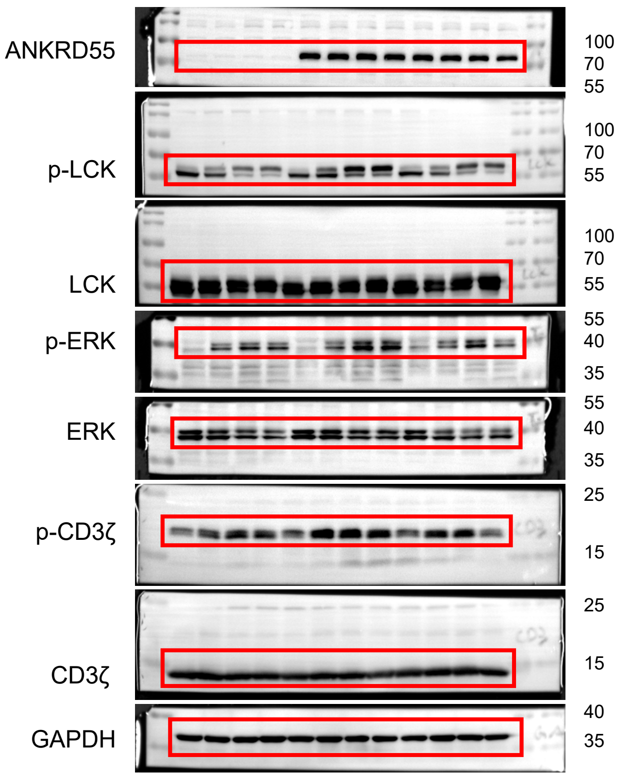

Figure S6B

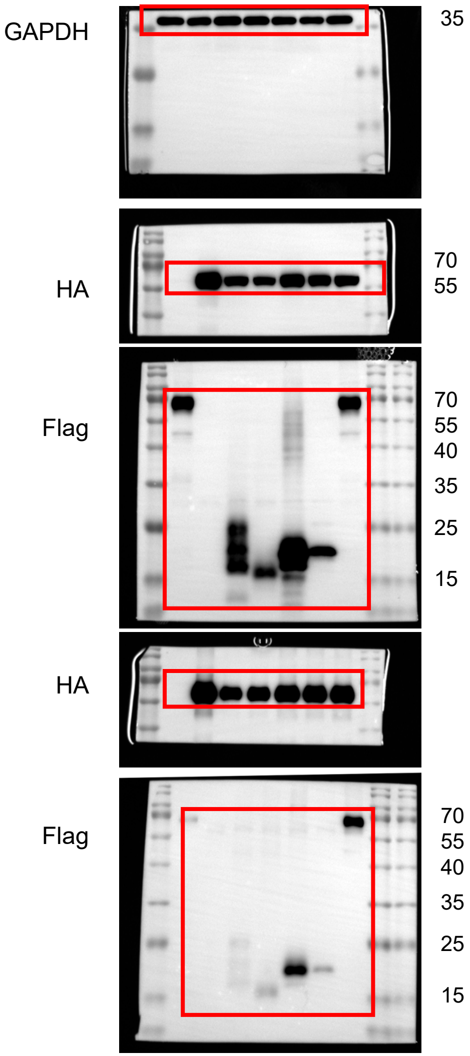

Figure S6C

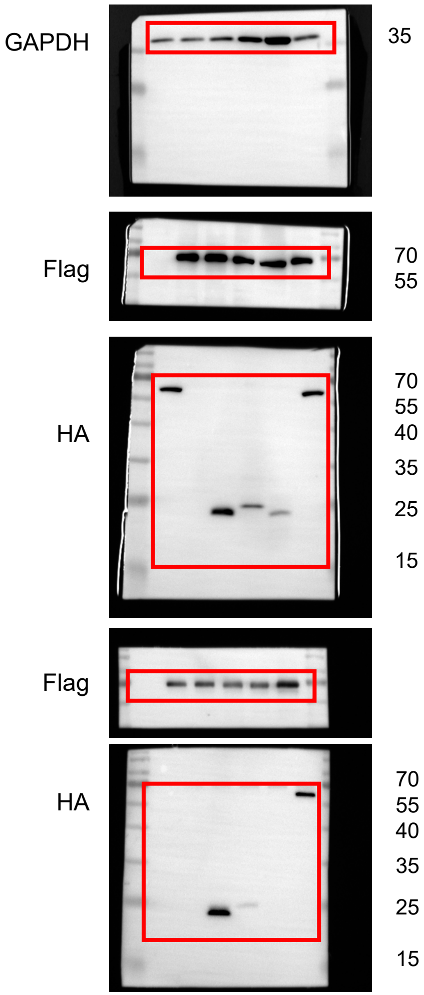

Figure S7A

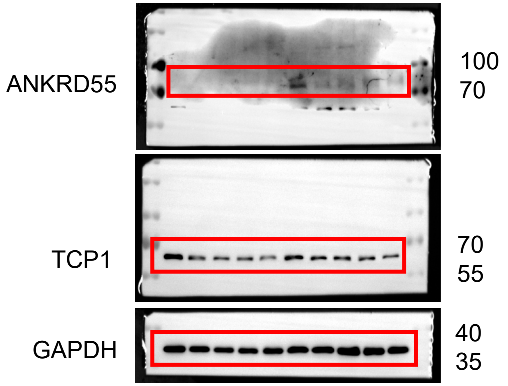

Figure S7B

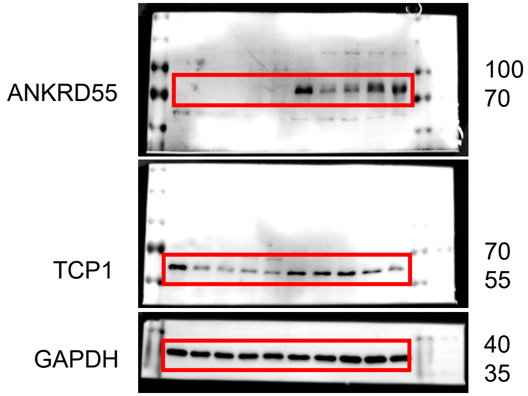

Figure S7C

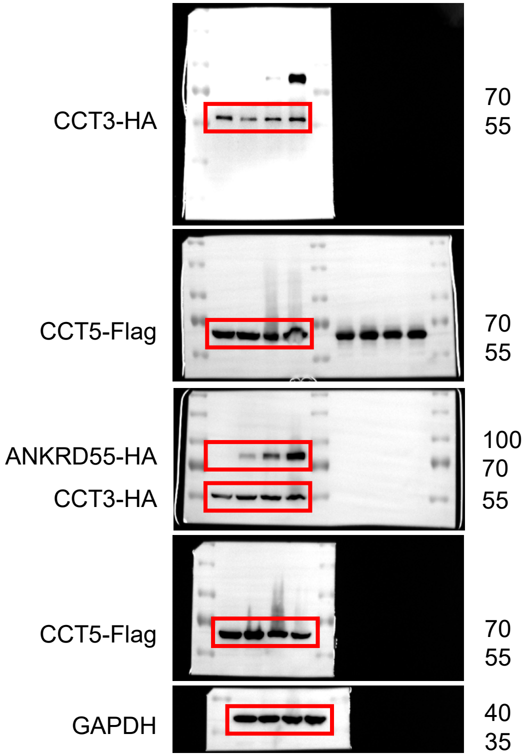

Figure S7D

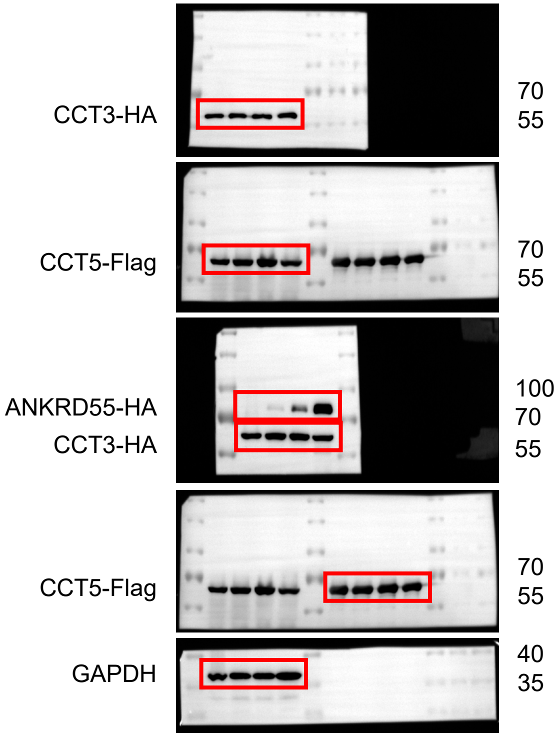

Figure S7E

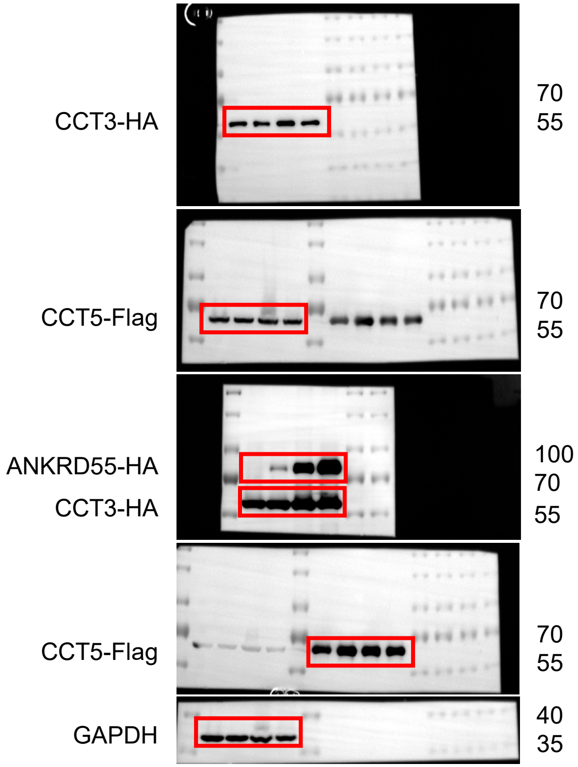

Supplement: Unedited blot and gel images [file jci-135-195214-s206.pdf]
